# The effect of zofenopril on the cardiovascular system of spontaneously hypertensive rats treated with the ACE2 inhibitor MLN-4760

**DOI:** 10.1186/s40659-023-00466-x

**Published:** 2023-10-25

**Authors:** Sona Cacanyiova, Martina Cebova, Fedor Simko, Tomas Baka, Iveta Bernatova, Michal Kluknavsky, Stefan Zorad, Katarina Krskova, Ezgi Shaman, Anna Zemancikova, Andrej Barta, Basak G. Aydemir, Andrea Berenyiova

**Affiliations:** 1 Centre of Experimental Medicine, Institute of Normal and Pathological Physiology, Slovak Academy of Sciences, Bratislava, Slovakia; 2 Institute of Experimental Endocrinology, Biomedical Research Center, Slovak Academy of Sciences, Bratislava, Slovakia; 3 Institute of Pathophysiology, Faculty of Medicine, Comenius University, Bratislava, Slovakia; 4 3rd Department of Internal Medicine, Faculty of Medicine, Comenius University, Bratislava, Slovakia

**Keywords:** Essential Hypertension, ACE2 inhibitor, Zofenopril, SARS-CoV-2, Cardiac function, Vasoactivity, Hydrogen sulfide, Nitric oxide, Mas receptor

## Abstract

**Background:**

Angiotensin converting enzyme 2 (ACE2) plays a crucial role in the infection cycle of SARS-CoV-2 responsible for formation of COVID-19 pandemic. In the cardiovascular system, the virus enters the cells by binding to the transmembrane form of ACE2 causing detrimental effects especially in individuals with developed hypertension or heart disease. Zofenopril, a H_2_S-releasing angiotensin-converting enzyme inhibitor (ACEI), has been shown to be effective in the treatment of patients with essential hypertension; however, in conditions of ACE2 inhibition its potential beneficial effect has not been investigated yet. Therefore, the aim of the study was to determine the effect of zofenopril on the cardiovascular system of spontaneously hypertensive rats, an animal model of human essential hypertension and heart failure, under conditions of ACE2 inhibition induced by the administration of the specific inhibitor MLN-4760 (MLN).

**Results:**

Zofenopril reduced MLN-increased visceral fat to body weight ratio although no changes in systolic blood pressure were recorded. Zofenopril administration resulted in a favorable increase in left ventricle ejection fraction and improvement of diastolic function regardless of ACE2 inhibition, which was associated with increased H_2_S levels in plasma and heart tissue. Similarly, the acute hypotensive responses induced by acetylcholine, L-NAME (NOsynthase inhibitor) and captopril (ACEI) were comparable after zofenopril administration independently from ACE2 inhibition. Although simultaneous treatment with zofenopril and MLN led to increased thoracic aorta vasorelaxation, zofenopril increased the NO component equally regardless of MLN treatment, which was associated with increased NO-synthase activity in aorta and left ventricle. Moreover, unlike in control rats, the endogenous H_2_S participated in maintaining of aortic endothelial function in MLN-treated rats and the treatment with zofenopril had no impact on this effect.

**Conclusions:**

Zofenopril treatment reduced MLN-induced adiposity and improved cardiac function regardless of ACE2 inhibition. Although the concomitant MLN and zofenopril treatment increased thoracic aorta vasorelaxation capacity, zofenopril increased the participation of H_2_S and NO in the maintenance of endothelial function independently from ACE2 inhibition. Our results confirmed that the beneficial effects of zofenopril were not affected by ACE2 inhibition, moreover, we assume that ACE2 inhibition itself can lead to the activation of cardiovascular compensatory mechanisms associated with Mas receptor, nitrous and sulfide signaling.

## Introduction

Angiotensin-converting enzyme 2 (ACE2) plays a key role in the infectious cycle of severe acute respiratory syndrome coronavirus 2 (SARS-CoV-2), which caused the coronavirus disease 2019 (COVID-19) pandemic. In general, there are two forms of ACE2, soluble and full-length transmembrane, anchored with an extracellular enzymatically active domain, which is the receptor for the spike protein of SARS-CoV-2 [1]. In uninfected cells, the main product of ACE2, Ang 1–7, binds to Mas receptors within the cardiovascular system and induces essential protective effects through its anti-inflammatory, antiproliferative-antifibrotic and antioxidant actions [2, 3]. However, dysfunction of the ACE2/Ang1-7/Ang1-9/Mas receptor pathway in the vasculature was shown to promote heart and vessel inflammation, structural rebuilding of the vasculature and endothelium-dependent or endothelium-independent dysfunction, including accelerated thrombogenesis [4, 5]. Thus, we could assume that the dysfunction of ACE2-mediated mechanisms could be detrimental, especially in individuals with preexisting cardiovascular complications. In our recent study, we imitated the assumed inhibition of ACE2-mediated signaling using a low dose of the ACE2 inhibitor MLN-4760 (MLN) with the aim of showing to what degree the inhibition of ACE2 is detrimental to the cardiovascular system of hypertensive rats. We found that in spontaneously hypertensive rats (SHRs), a model mimicking human essential hypertension, MLN induced detrimental effects on adiposity, the function of small arteries and angiogenesis, as well as a reduction in the ACE inhibitor (ACEI)-induced hypotensive effect. However, in the thoracic aorta, compensatory mechanisms involving accentuation of NO and H_2_S signaling have been described, thus pointing to the complex action of ACE2 inhibitor [6].

Commonly used antihypertensive drugs, especially ACEIs and angiotensin receptor type I blockers (ARBs), were shown to affect the ACE2-related pathway in the sense of ACE2 upregulation [7]. Recommendations regarding the application of ACEIs or ARBs during COVID-19 are currently actively debated; however, a number of analyses have shown that patients with cardiovascular disorders are not endangered by ACEI/ARB treatment during COVID-19 or may even benefit from it [8–10]. Sulfhydrylated ACEIs, especially zofenopril (ZOFE), have been shown to provide additional benefits in some indications compared to other ACE inhibitors. For instance, ZOFE improved the clinical outcomes of patients with various cardiovascular diseases, such as acute myocardial infarction [11], and ZOFE was also of conceivable benefit in the treatment of endothelial dysfunction [12–14]. Moreover, Bucci et al. [15] declared that ZOFE improved vascular function by potentiating the hydrogen sulfide (H_2_S) pathway in SHRs. H_2_S could interact with individual parts of the RAS [16, 17]. Moreover, H_2_S treatment attenuated atherosclerosis in a partially ligated carotid artery mouse model by stimulating ACE2 expression, while the ACE2 inhibitor MLN abolished the antiatherosclerotic effect of H_2_S [18]. In contrast, the administration of an H_2_S donor inhibited ACE expression in the kidney from SHR and prevented the development of hypertension [19]. By blocking the RAS, H_2_S has also been shown to improve endothelial function and myocardial remodeling in both renovascular hypertensive rats and streptozotocin-induced diabetic rats [20]. Moreover, Shi et al. [21] demonstrated that exogenous administration of an H_2_S donor markedly ameliorated left ventricular remodeling and cardiac fibrosis in SHR, indicating that H_2_S-releasing ACEI ZOFE could be effective in the treatment of cardiovascular alterations associated with essential hypertension; however, the impact of ACE2 remains unknown. The main objective of the present study was to determine the effect of ACEI treatment with ZOFE on the cardiovascular system in conditions of arterial hypertension and ACE2 inhibition using adult SHR. We aimed to evaluate changes in blood pressure, plasma RAS, adiposity, cardiac function, and thoracic aorta vasorelaxant properties with a specific focus on the role of NO, H_2_S and Mas receptor-mediated pathways.

## Materials and methods

### Experimental model

Sixteen- to eighteen-week-old male spontaneously hypertensive rats (SHRs, *n* = 80) were divided into four experimental groups: the control (SHR; *n* = 20), MLN-4760-treated (SHR + MLN; *n* = 20), zofenopril-treated (SHR + ZOFE; *n* = 20), and MLN-4760 and zofenopril-treated groups (SHR + MLN + ZOFE; n = 20). Each experimental group was divided into two separate subgroups: one for the realization of in vivo (echocardiography, integrated blood pressure response (*n* = 8) and one for in vitro studies (vasoactive responses and biochemical analyses, *n* = 12). The samples of randomly selected 8–10 rats were used in the study.

The specific ACE2 inhibitor MLN-4760 (MedChemExpress, Monmouth Junction, NJ, USA) was administered using Alzet® miniosmotic pumps (Durect™, Cupertino, CA, USA), Model 2002, with a pumping rate of 0.5 µL/h for 14 days at a dose of 1 mg/kg/day dissolved in 10% dimethyl sulfoxide (DMSO) in isotonic saline (sodium chloride 0.9% Braun intravenous solution for infusion; 308 mOsm/L; 250 mL; B. Braun Melsungen AG, Melsungen, Germany) in the SHR + MLN and SHR + MLN + ZOFE groups. In the SHR and SHR + ZOFE groups, miniosmotic pumps were filled with 10% DMSO in isotonic saline. All pumps were implanted subcutaneously on the dorsum of rats under isoflurane inhalation (2.5-3%) anesthesia in aseptic conditions. The cut was closed by three stitches of braided nonabsorbable silk suture (SMI, St. Vith, Belgium). The SHR + ZOFE and SHR + MLN + ZOFE groups received Zofenopril calcium (AdooQ Bioscience LLC, Irvine, CA, USA) that was administered p.o. (mixed with chow) at a dose of 10 mg/kg/day for 10 days. Treatment with ZOFE started on the 5^th^ day of MLN administration in the SHR + ZOFE and SHR + MLN + ZOFE groups to determine its function in rats in which ACE2 function was disrupted.

### General parameters and blood pressure determination

Basal and final body weights (BWs) were determined 1 day before minipump implantation and on the last day of treatment, respectively. The changes in BW in each group were expressed as a percentage of the weight gain during the treatments (Δ BW%). Systolic blood pressure (SBP) was measured using tail-cuff plethysmography (MRBP, IITC Life Science Inc., Los Angeles, CA, USA). The SBP was expressed as the percent difference between the SBP values at the beginning and end of the treatments (Δ SBP%). The basal SBP was measured 3 days before minipump implantation. The measurement of the final blood pressure was performed on Day 12 in subgroups for in vivo studies or Day 13 in subgroups for in vitro studies. Five measurements were performed on each rat, and SBP was calculated as the average of the last four measurements.

### In vivo studies

#### Echocardiography

Transthoracic echocardiography was performed on eight animals per group (the in vivo subgroup of the experiment) at the end of the experiment using a 14-MHz matrix probe (M12 L) coupled with a GE Medical Vivid 7 Dimension System (GE Medical Systems CZ Ltd., Prague, Czech Republic), as described previously [22, 23]. For general anesthesia, a 2.5% inspiratory concentration of isoflurane at a flow rate of 2 L/min during spontaneous breathing was used throughout the protocol. The rat was placed in the supine position on a warming pad (38 °C), and the transthoracic wall was shaved. The body temperature and heart rate were continuously monitored. To assess the systolic function of the left ventricle (LV), the LV end-systolic and end-diastolic internal diameters were measured from the anatomical M-mode images in a long-axis view using the leading-edge method. The left ventricular fractional shortening (LVFS) and ejection fraction (LVEF, using the Teichholz formula) were subsequently determined. To assess the diastolic function of the LV, the diastolic transmitral peak early (E) and late (A) filling velocities were measured from the two-dimensionally guided Doppler spectra of mitral inflow in the apical four-chamber view, and the E/A ratio was subsequently calculated. Tissue Doppler imaging from the apical four-chamber view was used to determine the maximal velocities of the early (Em) and late (Am) diastolic wall movement waves at the level of the septal mitral annulus, and the E/Em ratio was subsequently calculated. Echocardiographic assessment of LV function was performed by an experienced blinded echocardiographer. Measurements were averaged over three consecutive cardiac cycles.

#### Integrated blood pressure response

The in vivo measurement of blood pressure was performed under inhalation anesthesia using isoflurane (2.5%, O_2_-1.5 L/min), which was continually maintained during the experiment, the body temperature was constantly kept at 38 °C by a warming pad. For the application of vasoactive substances, the right jugular vein was cannulated, and heparin sulfate (25 IU, 100 µL) was administered to prevent coagulation. Then, a pressure sensor connected to a pressure transducer (FOP-LS-PT9-10, FISO Technologies, Quebec, Canada) was placed in the right carotid artery. Later, the basal BP was left to stabilize within 15–20 min, and the vasoactive compounds dissolved in 100 µL saline were administered into the jugular vein over a 10 s period. The integrated pressure responses were induced by noradrenaline (NA, 1 µg/kg; agonist of adrenergic receptors; Zentiva, Prague, Czech Republic), acetylcholine (Ach, 1 µg/kg; agonist of muscarinic receptors), captopril (10 mg/kg; angiotensin-converting enzyme inhibitor), bismuth(III) subsalicylate (BSC, 0.25 µg/kg; hydrogen sulfide scavenger), and N^G^-nitro-L-arginine methylester (L-NAME, 30 mg/kg; NO synthase inhibitor). The responses of blood pressure were expressed as the mean arterial BP (MAP). The changes in MAP (ΔMAP in mmHg) stimulated by Ach and NA were measured as the difference between values of maximal response after the addition of the compound (blood pressure increase or decrease) and basal MAP (immediately before the addition of the compound). The responses stimulated by captopril, BSC, and L-NAME, which generated long-lasting responses, were evaluated 10 min after their injection. The response to Ach (1 µg/kg) was realized again after a 15 min pretreatment with L-NAME (30 mg/kg). Stock solutions of all compounds were prepared freshly on the day of the experiments and used within a few hours. The recorded analog signal was digitalized, and DEWESoft 6.6.7 software (DEWETRON, Prague, Czech Republic) was used for data collection and further analysis.

### Ex vivo studies

On Day 14 of the treatment, the rats were sacrificed by decapitation after brief CO_2_ anesthesia. Trunk blood was collected into preprepared heparinized tubes (140 UI/5 mL) and then centrifuged (850× g, 10 min, 4 °C, Centrifuge 5430 R, Eppendorf, Hamburg, Germany). Plasma was aliquoted and stored at -80 °C until the analysis of H_2_S and angiotensins. Tissue samples (heart, visceral fat - retroperitoneal plus epididymal fat, aortic tissue) were weighed, rapidly frozen in liquid nitrogen and stored at -80 °C for further analyses. The thoracic aorta (TA) was isolated for the in vitro functional study, which was performed on the day of sacrifice.

#### Determination of H_2_S concentration in plasma and heart tissue

The H_2_S concentration in plasma and heart tissue was measured using a methylene blue assay as described in detail previously [24, 25]. Briefly, 75 µL of the plasma samples was added to 0.1 mol/L potassium phosphate buffer (325 µL), and then this mixture was combined with reaction buffer (total volume 500 µL) containing pyridoxal-5-phosphate and L-cysteine. The heart tissue was homogenized with lysis buffer including sodium orthovanadate and protease inhibitor. The protein concentration was determined by the Lowry method. The absorbance was measured at 650 nm via a spectrophotometer (NanoDrop™ 2000/2000c Spectrophotometers, Thermo Fisher Scientific, Waltham, MA USA). All standards and samples were assayed in duplicate using a 96-well plate. The H_2_S concentration was calculated against a calibration curve of NaHS (3.9–250 µmol/L). The results of plasma H_2_S concentration are given in µmol/L, and the results of heart tissue are determined as nmol/g of protein.

#### Analysis of angiotensins

The equilibrium concentration of angiotensin (1–10) (Ang I), angiotensin (1–8) (Ang II), angiotensin (1–7), and angiotensin (1–5) were identified using liquid chromatography-tandem mass spectrometry (LC-MS/MS) in heparinized plasma samples as described previously [26]. The basis of the ex vivo analysis is to determine the established equilibrium concentration between angiotensin peptide formation and elimination, and it considers all plasma RAS soluble factors of angiotensin production/elimination in the condition of RAS activity. Therefore, the total alternative RAS activity can be calculated relying on this analysis as a ratio of (Ang 1–7 + Ang 1–5)/(Ang I + Ang II + Ang 1–7 + Ang 1–5) as well as ACE activity as a ratio of Ang II/Ang I. The renin activity is expressed as sum of Ang I and Ang II concentrations.

### Vasoactive responses of thoracic aorta

The segment of thoracic aorta (TA) was removed from the intact aorta beginning 5 mm below the aortic arch and ending before a diaphragm and cut into 5 mm length rings. Setting such a length of the specimen is suitable for the used type of tissue holder and allows optimal and reproducible vasoactive responses to be recorded. The TA was carefully cleaned, its connective and adipose tissue was removed while avoiding impairment of the endothelium. Then, TA was placed vertically fixed between two stainless steel wire triangles, and inserted into a 20-mL organ bath with Krebs solution (oxygenated with 95% O_2_ and 5% CO_2_, 37 °C) containing 118 mmol/L NaCl, 5 mmol/L KCl, 25 mmol/L NaHCO_3_, 1.2 mmol/L MgSO_4_, 1.2 mmol/L KH_2_PO_4_, 2.5 mmol/L CaCl_2_, 11 mmol/L glucose, and 0.032 mmol/L Ca-Na_2_EDTA. The bottom triangles were fixed, and the upper triangles were connected with a sensor of isometric tension (FSG-01, MDE GmbH, Budapest, Hungary), thereby recording the vasoactive changes via an NI USB-6221 AD converter (MDE GmbH, Budapest, Hungary) and S.P.E.L. Advanced Kymograph software (MDE GmbH, Budapest, Hungary). To set the optimal diameter of thoracic aorta segment close to the in vivo values, a series of preliminary experiments was carried out. Different values of resting pretension, from the lowest to the highest, were applied to rings of the thoracic aorta isolated from SHR and the rings were exposed to increasing concentrations of noradrenaline inducing a concentration-dependent contractile response. The value of resting tension (1 g) at which the largest contractile response was repeatedly recorded was applicated for arterial rings used in this study. A resting tension of 1 g was carried out on each TA ring to eliminate any nonspecific stress relaxation between 45 and 60 min until the arteries were stabilized.

Single concentrations of NA (10^− 6^ mol/L) and ACh (10^− 5^ mol/L) were added to test the integrity of the arterial wall (the contractile abilities and the integrity of the endothelium). The endothelium-dependent relaxant response was evaluated on the NA-precontracted (10^− 6^ mol/L) TA rings. Increasing concentrations of exogenous ACh (10^− 10^-10^− 5^ mol/L) were cumulatively applied after achieving a stable plateau. The ratio of vasorelaxation was determined as a percentage of the maximal contraction stimulated by NA.

Next, we examined the participation of certain signaling pathways in the vasoactive responses of the TA. To investigate the participation of Mas receptors in vasoactive responses, a selective Mas receptor inhibitor, A-799 trifluoroacetate salt, was used (10^− 5^ mol/L). To specify the role of the endogenous NO pathway, the rings of TA were incubated with a nonspecific inhibitor of NO synthase, N^G^-nitro-L-arginine methyl ester (L-NAME, 10^− 5^ mol/L). The H_2_S scavenger bismuth(III) subsalicylate (BSC, 10^− 5^ mol/L) was used to evaluate the participation of H_2_S in the vasoactive responses. To determine the effect on endothelium-derived relaxation, all mentioned compounds were acutely incubated for 20 min in the organ bath prior to the addition of NA (10^− 6^ mol/L) and ACh (10^− 10^-10^− 5^ mol/L).

### Total NO synthase activity

Total NOS activity was evaluated in 10% of aorta homogenates and 20% of left ventricle homogenates by following the previous instructions [27]. The Quanta Smart Tri-Carb Liquid Scintillation Analyzer (TriCarb, Packard, UK) was used for measuring [3 H]-L-citrulline formation from [3 H]-L-arginine (MP Biochemicals, CA, USA). NOS activity was expressed as picokatal per gram of protein (pkat/g protein).

### Superoxide production measurement

The production of superoxide was measured in samples of the left heart ventricle (~ 10–20 mg) and thoracic aorta (~ 5–15 mg, ring segments cleaned of surrounding adipose and connective tissue) using lucigenin-enhanced chemiluminescence. Freshly collected tissue samples (two samples from each tissue) were placed into Eppendorf test tubes with ice-cold modified Krebs-Henseleit (KH) solution (KH concentration in mmol/L: 119 NaCl, 4.7 KCl, 1.17 MgSO_4_·7H2O, 25 NaHCO_3_, 1.18 KH_2_PO_4_, 0.03 Na_2_ EDTA, 2.5 CaCl_2_·2H_2_O, 5.5 glucose). After all tissue samples were obtained, a freshly prepared stock solution of lucigenin (5 mmol/L, dissolved in ice-cold KH) was prepared. From a freshly prepared lucigenin stock solution, a lucigenin measuring solution was prepared by diluting the stock solution with pneumoxide-bubbled KH (5% CO_2_ and 95% O_2_, pH 7.4, temperature 37 °C) to a final concentration of 50 µmol/L. Samples were gradually placed into 1.5 mL Eppendorf tubes containing 1 mL of pneumoxide-bubbled KH solution, and preincubated in the dark at 37 °C for 18 min. At the same time, 1.5 mL Eppendorf test tubes containing 1 mL of lucigenin measuring solution were gradually preincubated under the same conditions.

After preincubation, the tissue samples were transferred into preincubated Eppendorf test tubes containing lucigenin measuring solution, and chemiluminescence was immediately measured in 10 s intervals for 3 min (18 measurements in total) using a GloMax® 20/20 Luminometer (Promega, Southampton, UK). Chemiluminescence was also measured in a blank sample containing lucigenin measurement solution alone. The average chemiluminescence value was calculated from the last 12 measurements for each tissue sample (as well as the blank sample) in doublets. Then, the chemiluminiscence of the blank was subtracted from the chemiluminescence of the tissue samples, and doublets were averaged to obtain the final chemiluminescence level. Superoxide production was expressed as relative chemiluminescence units per mg of tissue sample (RLU/mg).

### RNA isolation and determination of gene expression

The gene expression levels of neuronal nitric oxide synthase (*Nos1*), inducible NOS (*Nos2*), endothelial NOS (*Nos3*), and 60 S ribosomal protein RPL10a (*Rpl10a)* in the aorta were determined by using two-step reverse transcription quantitative polymerase chain reaction (RT-qPCR). The total RNA of the aorta was isolated using the PureZOL™ RNA Isolation Reagent (Bio-Rad, Hercules, CA, USA) according to the manufacturer’s protocols. The amount and purity of total isolated RNA was spectrophotometrically quantified at 260/280 and 260/230 nm using a NanoDrop spectrophotometer (Thermo Scientific, Waltham, MA, USA). Reverse transcription was performed using 1 µg of total RNA from each sample using an Eppendorf Mastercycler (Hamburg, Germany) and an iScript-Reverse Transcription Supermix (Bio-Rad, Hercules, CA, USA) according to the manufacturer’s protocols. Gene-specific primers were designed using the PubMed program (Primer-BLAST) and database (Gene). The DNA sequences and melting temperature of the primers used, the size of the amplicons and the reference numbers of the templates are described in Table 1. The PCRs were conducted in a final volume of 20 µL containing 2 µL of 5-fold diluted template cDNA, 10 µL SsoAdvanced mix (SsoAdvanced Universal SYBR Green Supermix, Bio-Rad, Hercules, CA, USA), 1.5 µL of both forward and reverse primers (Metabion, Germany, 4 µmol/L), and 5 µL RNase free water (Sigma–Aldrich, Germany) in a final volume of 20 µL. The thermal cycling conditions were as follows: (1) 95 °C for 30 s, (2) 40 cycles consisting of *(a)* 95 °C for 10 s, and *(b)* an optimal annealing temperature (depending on the selected primer, see Table 1) for 20 s. Finally, melt curves for amplicon analyses were constructed at 60–95 °C, 5 s/1°C. RT‒qPCR was performed using a CFX96 Real-Time PCR (Bio-Rad, Hercules, CA, USA) detection system and evaluated by Bio-Rad CFX Manager software 2.0 (Bio-Rad, Hercules, CA, USA). The expression of each gene was determined in 8 rats. The quantities (Ct values) of target genes were normalized to the quantities (Ct) of the housekeeping gene (*Rpl10a*). Relative mRNA expression was calculated using the 2^−ΔΔCt^ method. For *Ace2* and *Mas1*, RNA (n = 7–12) was separated from deep-frozen abdominal aorta tissue samples (-80 °C) by application of a Minilys personal homogenizer (Bertin Technologies SAS, Montigny-le-Bretonneux, France) and a RNeasy Fibrous Tissue Mini Kit (Qiagen, Valencia, CA, USA) following the manufacturer´s protocol. Reverse transcription of isolated RNA was performed by a High-Capacity cDNA Reverse Transcription Kit (Thermo Fisher Scientific, Waltham, MA, USA) by following the manufacturer’s instructions. RT-qPCR was performed in FastStart Universal SYBR Green Master mix solution (Roche, Indianapolis, IN) and a QuantStudio™ 5 Real-Time PCR System (Applied Biosystems, Thermo Fisher Scientific, Waltham, MA, USA). Rat-specific primer pairs are shown in Table 1. Data from mRNA levels were normalized to the expression of the housekeeping gene TATA box binding protein (*Tbp*), which was not changed by the treatment.

**Table 1:** Used primer pairs

Gene	Forward primer	Reverse primer	Tm (°C)	Amplicon size (bp)
*Nos1* (NM_052799.1)	GCA GAG GCC GTC AAG TTC T	GAG AAT GGT CGC CTT GAC CC	60	72
*Nos2* (NM_012611.3)	AAA CGC TAC ACT TCC AAC GC	TGC TGA GAG CTT TGT TGA GGT C	59	91
*Nos3* (NM_021838.2)	GAT CCC CCG GAG AAT GGA GA	TCG GAT TTT GTA ACT CTT GTG CT	60	105
*Cbs* (NM_012522.2)	ATG GTG ACT CTC GGG AAC ATG	AGG TGG ATC GGC TTG AAC TG	59	104
*Ace2* (NM_001012006.2)	TCA GAG CTG GGA TGC AGA AA	GGC TCA GTC AGC ATG GAG TTT	60	111
*Mas1* NM_012757.2)	TTC ATA GCC ATC CTC AGC TTC TTG	GTT CTT CCG TAT CTT CAC CAC CAA	60	84
*Rpl10a* (NM_031065.1)	TCC ACC TGG CTG TCA ACT TC	GGC AGC AAC GAG GTT TAT TGG	60	134

Abbreviations: Tm, melting temperature; bp, base pair of DNA

### Western blotting

Protein expression levels of H_2_S producing enzymes - cystathionine-γ-lyase (CSE) and cystathionine-β-synthase (CBS), ACE2, the Mas receptor, and all NOS isoforms were evaluated in the aorta and LV by Western blot analysis. First, tissue samples were homogenized and then centrifuged at G 17 608,5 and at 4 °C for 20 min. For homogenization, lysis buffer containing 0.05 mmol/L Tris and protease inhibitor cocktail was used. A Lowry assay was used to calculate the protein concentrations of the supernatant [28]. The equal amounts of protein (50 µg) were subjected to SDS-PAGE using 12% gels and transferred to nitrocellulose membranes. 5% nonfat milk in TBST (Tris-buffer solution, pH 7.6, containing 0.1% Tween-20) was used to block the membranes for 1 h at room temperature. After washing the membranes in TBST, the following primary antibodies were applied: rabbit polyclonal anti-endothelial NOS, anti-neuronal NOS, anti-GAPDH and anti-β-actin (Abcam, Cambridge, UK); rabbit polyclonal anti-inducible NOS (Bio-Rad, Inc., Hercules, CA, USA); rabbit polyclonal anti-Ang1-7, Mas receptor (Alomone Labs, Jerusalem, Israel); rabbit polyclonal anti-CBS and mouse monoclonal anti-CSE antibodies (Proteintech, Manchester, UK); and rabbit monoclonal anti-ACE2 (Invitrogen, Waltham, MA, USA) overnight at 4 °C. Primary antibody binding was determined using a secondary horseradish peroxidase-conjugated antirabbit antibody (Abcam, Cambridge, UK) or horseradish peroxidase-conjugated anti-mouse antibody (Cell Signaling Technology, Danvers, MA, USA) at room temperature for 2 h. An enhanced chemiluminescence system (ECL, Amersham, UK) was used for band intensity visualization. The ChemiDocTM Touch Imagine System (Image LabTM Touch software, Bio-Rad, Inc., Hercules, CA, USA) was used for quantification of the bands, with β-actin or GAPDH used as an internal loading control in aorta and LV, respectively.

### Fluorescent staining of proteins

The distribution of the enzymes (CSE, CBS) was stained using 10 μm thick cryosections of TA (Tissue-Tek O.C.T. compound, Leica Biosystems, Deer Park, IL, USA). The sections were mounted onto SUPERFROST PLUS adhesion microscope slides (Epredia™, Fisher Scientific, Waltham, MA, USA) and fixed in 4% paraformaldehyde. Then, the slides were immersed in 0.1% Triton to permeabilize the membrane and blocked in 1% bovine serum albumin (BSA), both diluted with phosphate-buffered saline (PBS, pH = 7.4). The samples were incubated at 4 °C (overnight) with primary antibodies: mouse monoclonal CSE antibody and rabbit CBS polyclonal antibody (both diluted 1:100 in 1% BSA, Proteintech, Manchester, UK). The next day, after washing in PBS, the slides were incubated for 90 min in the dark with secondary antibodies: green FITC-fluorescein isothiocyanate for CBS (diluted: 1:2000, Abcam, Cambridge, UK) and red Alexa Fluor 532 for CSE (diluted 1:2000, Thermo Fisher Scientific, Waltham, MA, USA). To reduce the nonspecific signals (autofluorescence of the laminae in the media), staining with 0.25% sudan black B (in 70% isopropyl alcohol) was used. After 90 min of incubation in the dark, the slides were washed first with 70% isopropyl alcohol and 3x in PBS, and tissues were mounted in Vectashield antifade mounting medium containing 4´,6´- diamidino-2-phenylindole (DAPI) for staining of the nuclei (Vector Laboratories Inc., Burlingame, CA, USA). The samples were captured by confocal microscopy (NikonC4) with a Nikon APO-TIRF 60/1.49 oil objective and visualized by NIS-Elements software.

### Statistical analysis

The group size was calculated by a priori analysis using G*Power software v3.1 [29] based on the expected effect of ACE inhibition on blood pressure, using the following values: effect size 0.55, α-error 0.05, power 0.85. Total sample size was calculated to 32 (n = 8/group). This number was increased to 40 because endothelial damage during in vitro experiment or death of the rat during the in vivo experiment may occur. Thus, the final number of rats in individual parameters was 8–10/group. The normality of the data was tested by the Shapiro-Wilks test. Three-way analysis of variance (ANOVA) for repeated measurements with the Bonferroni post hoc test was used to evaluate vasoactive responses. To evaluate cardiovascular and biochemical data, NO synthase activity, superoxide production, H_2_S concentrations, gene, and protein expression, and cardiac (systolic, diastolic) function, 2-way ANOVA was used. Data were analyzed using OriginPro (OriginLab Corporation, Northampton, MA, USA), GraphPad Prism 7.0 (GraphPad Software, Inc., La Jolla, CA, USA) and Statistica 13.5 (StatSoft, Hamburg, Germany). The data are expressed as the mean ± S.E.M.

### Drugs

All the chemicals used in this study were purchased from Merck (Bratislava, Slovakia) unless stated otherwise.

## Results

### General characteristics of the experimental groups

#### Blood pressure and biometrical parameters

At the beginning of the MLN and ZOFE treatments, there was no difference in the values of SBP among the experimental groups (SHR: 161 ± 5.8 mmHg, SHR + MLN: 163 ± 5.5 mmHg, SHR + ZOFE: 155 ± 1.4 mmHg, SHR + MLN + ZOFE: 159 ± 5.5 mmHg). MLN treatment had no impact on SBP; similarly, ZOFE treatment, regardless of MLN treatment, had no impact on SBP (Table 2). The basal body weight of the rats was comparable between the groups as well (SHR: 292 ± 3.7 g, SHR + MLN: 296 ± 3.8 g, SHR + ZOFE: 290 ± 4.9 g, SHR + MLN + ZOFE: 294 ± 2.4 g). However, the rats treated with MLN gained significantly more weight than the control rats (p < 0.05). On the other hand, the ZOFE treatment decreased the weight gain compared to SHR (p < 0.01), and the same effect was observed in the SHR + MLN + ZOFE group compared to the SHR + MLN group (p < 0.001). We observed a significantly decreased heart weight and heart weight/body weight ratio in the SHR + MLN + ZOFE group compared to the SHR + MLN group (p < 0.05). The MLN treatment increased the weight of the visceral fat and the visceral fat weight body weight ratio (p < 0.05). Similarly, the plasma level of H_2_S was increased in the SHR + MLN group (p < 0.001), but ZOFE treatment either alone or in combination with MLN had no impact. However, ZOFE administration alone increased the basal and activated H_2_S concentrations in heart tissue (p < 0.001), like MLN (p < 0.001 and p < 0.01). Simultaneous MLN and ZOFE administration led to a decreased basal H_2_S concentration compared to the basal H_2_S concentration in the ZOFE group (p < 0.001); however, simultaneous MLN and ZOFE administration increased the activated H_2_S concentration even more than after treatment with MLN only (p < 0.01).

**Table 2:** General Characteristics of Experimental Animals

	SHR	SHR + MLN	SHR + ZOFE	SHR + MLN + ZOFE
**n**	20	20	20	20
**ΔSBP (%)**	5.87 ± 2.31	7.16 ± 2.83	-2.69 ± 2.04	6.65 ± 2.45
**ΔBW (%)**	6.05 ± 0.44	8.38 ± 0.45*	3.08 ± 0.8**	5 ± 0.71^+++^
**HW (g)**	1.12 ± 0.04	1.21 ± 0.03	1.1 ± 0.01	1.04 ± 0.03^+^
**HW/BW (mg/g)**	3.72 ± 0.14	3.78 ± 0.09	3.46 ± 0.03	3.4 ± 0.09^+^
**VF (g)**	7.83 ± 0.33	8.99 ± 0.3*	8.17 ± 0.39	8.07 ± 0.19
**VF/BW (mg/g)**	25.1 ± 0.86	28.1 ± 0.93*	25.59 ± 1.07	26.24 ± 0.5
**H**_**2**_ **S plasma (µM)**	11.89 ± 2.41	22.7 ± 8.48***	14.27 ± 3.65	20.23 ± 6.74**
**H**_**2**_ **S heart (nM/mg) – basal**	826 ± 25.99	1020.67 ± 41.33***	1818.37 ± 127.84***	1079.76 ± 18.89***^§§§^
**H**_**2**_ **S heart (nM/mg) – activated**	1062.67 ± 61.76	1534 ± 91.17**	2263.27 ± 223.52***	2020.24 ± 105.84***^++^

Abbreviations: ΔSBP – the changes in systolic blood pressure, ΔBW – body weight gain, HW – heart weight, HW/BW – ratio of heart weight to body weight, VF – weight of visceral fat, VF/BW – ratio of visceral fat to body weight, n – number of rats. SHR – spontaneously hypertensive rats, SHR + MLN – spontaneously hypertensive rats treated by MLN-4760, SHR + ZOFE – spontaneously hypertensive rats treated by zofenopril, SHR + MLN + ZOFE – spontaneously hypertensive rats treated by MLN-4760 and zofenopril. The results are presented as the mean ± S.E.M., and differences between groups were analyzed by two-way ANOVA, * p < 0.05, ** p < 0.01, *** p < 0.001 vs. SHR,^+^ p < 0.05, ^+++^p < 0.001 vs. SHR + MLN, ^§§§^ p < 0.001vs. SHR + ZOFE

#### Analysis of angiotensins in plasma

**Fig. 1:**
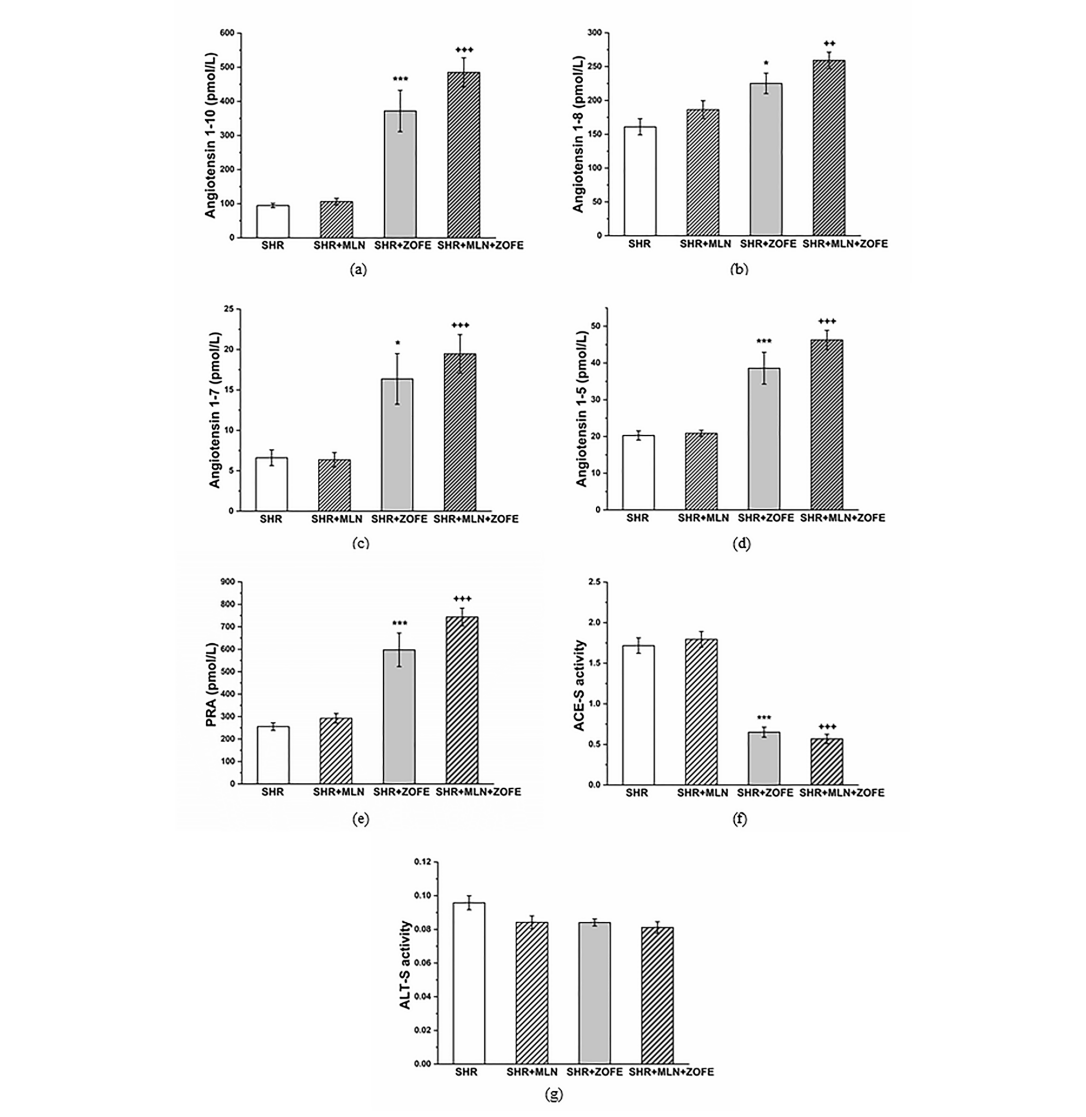
Equilibrium concentration of angiotensin peptides (**a**-**d**). The effect of MLN and ZOFE treatments on the equilibrium plasma renin activity (**e**) and activity of soluble RAS enzymes determined from angiotensin product/substrate (pmol/L/pmol/L) ratios in plasma (**f**,**g**). PRA – plasma renin activity, ACE-S – soluble angiotensin converting enzyme, ALT-S – total alternative renin angiotensin system activity, SHR – spontaneously hypertensive rats (n = 10), SHR + MLN – spontaneously hypertensive rats treated by MLN-4760 (n = 10), SHR + ZOFE – spontaneously hypertensive rats treated by zofenopril (n = 10), SHR + MLN + ZOFE – spontaneously hypertensive rats treated by MLN-4760 and zofenopril (n = 10). The results are presented as the mean ± S.E.M., and differences between groups were analyzed by two-way ANOVA, * p < 0.05, *** p < 0.001 vs. SHR, ^++^ p < 0.01, ^+++^ p < 0.001 vs. SHR + MLN

MLN treatment alone had no impact on the plasma levels of angiotensin 1–10, 1–8, 1–7 and 1–5; however, ZOFE administration, alone or in combination with MLN, increased the levels of all analyzed angiotensins (Fig. 1a-d). We observed a similar pattern in plasma renin activity (PRA, p < 0.001, Fig. 1e); however, in the SHR + ZOFE and SHR + MLN + ZOFE groups, the activity of soluble angiotensin converting enzyme (ACE activity, p < 0.001, Fig. 1f) was reduced, and all treatments had no effect on the total alternative renin angiotensin system activity (ALT-S, Fig. 1g).

### In vivo Studies

#### Echocardiography

**Fig. 2:**
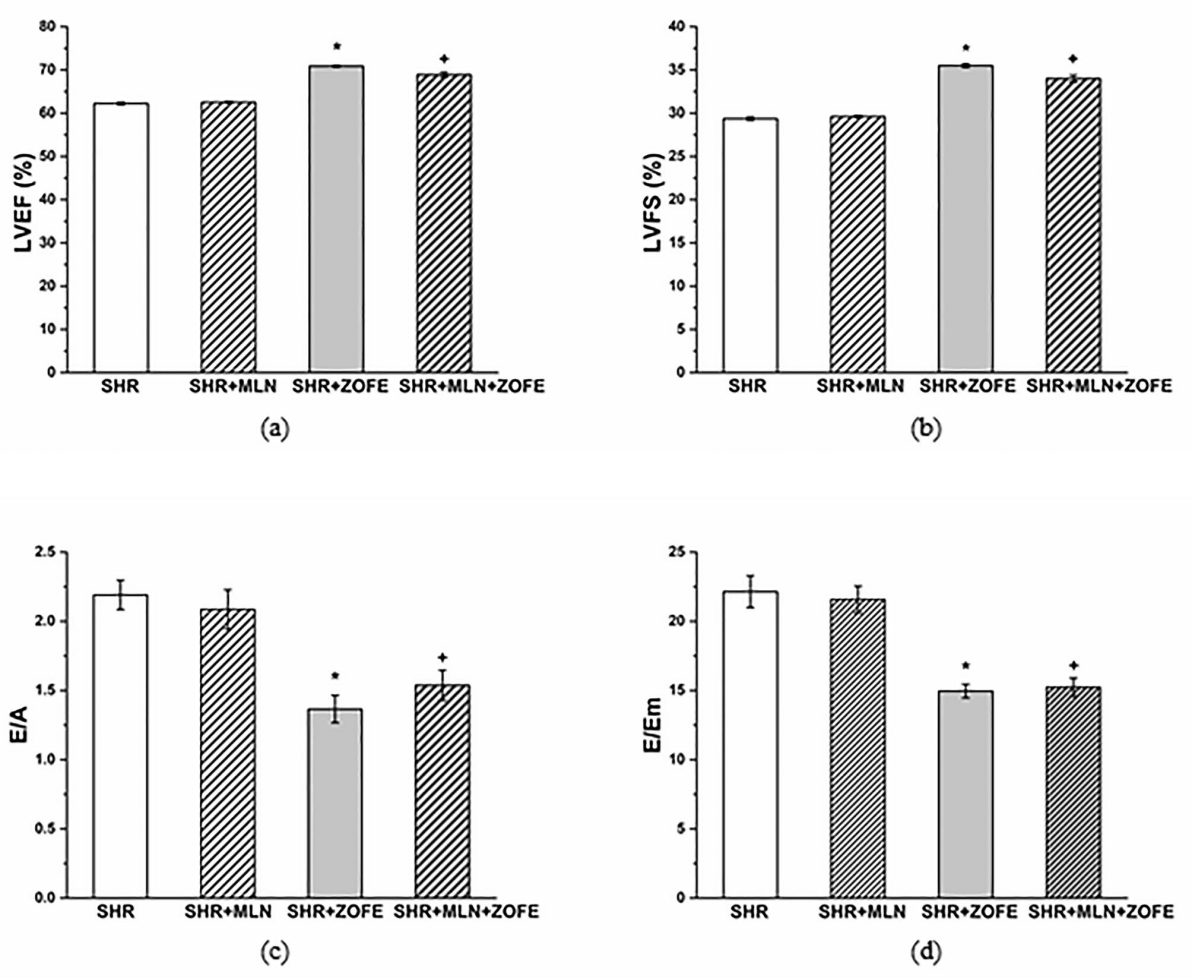
The effect of MLN and ZOFE treatment on left ventricular ejection fraction (LVEF) (**a**), left ventricular fractional shortening (LVFS) (**b**), the ratio of the diastolic transmitral peak early and late filling velocities (E/A ratio) (**c**), and the ratio of the diastolic transmitral peak early filling velocity and the maximal velocity of early diastolic wall movement wave at the level of mitral annulus (E/Em ratio) (**d**). SHR – spontaneously hypertensive rats (n = 8), SHR + MLN – spontaneously hypertensive rats treated by MLN-4760 (n = 8), SHR + ZOFE – spontaneously hypertensive rats treated by zofenopril (n = 8), SHR + MLN + ZOFE – spontaneously hypertensive rats treated by MLN-4760 and zofenopril (n = 8). The results are presented as the mean ± S.E.M., and differences between groups were analyzed by two-way ANOVA, * p < 0.05 vs. SHR, ^+^ p < 0.05 vs. SHR + MLN

The LVEF was 62.25 ± 0.31% in the SHR group, and MLN had no effect. ZOFE increased LVEF in both SHR and MLN-treated rats by 14% (p < 0.05) and 10% (p < 0.05), respectively (Fig. 2a). The LVFS was 29.37 ± 0.22% in the SHR group, and MLN had no effect. ZOFE increased LVFS in both SHR and MLN-treated rats by 21% (p < 0.05) and 15% (p < 0.05), respectively (Fig. 2b). The E/A ratio was 2.19 ± 0.11 in the SHR group, and MLN had no effect. ZOFE decreased the E/A ratio in both SHR and MLN-treated rats by 38% (p < 0.05) and 26%, (p < 0.05), respectively (Fig. 2c). The E/Em ratio was 22.14 ± 1.15 in the SHR group, and MLN had no effect. ZOFE decreased the E/Em ratio in both SHR and MLN-treated rats by 32% (p < 0.05) and 29% (p < 0.05), respectively (Fig. 2d).

#### Integrated pressure responses of the cardiovascular system

**Fig. 3:**
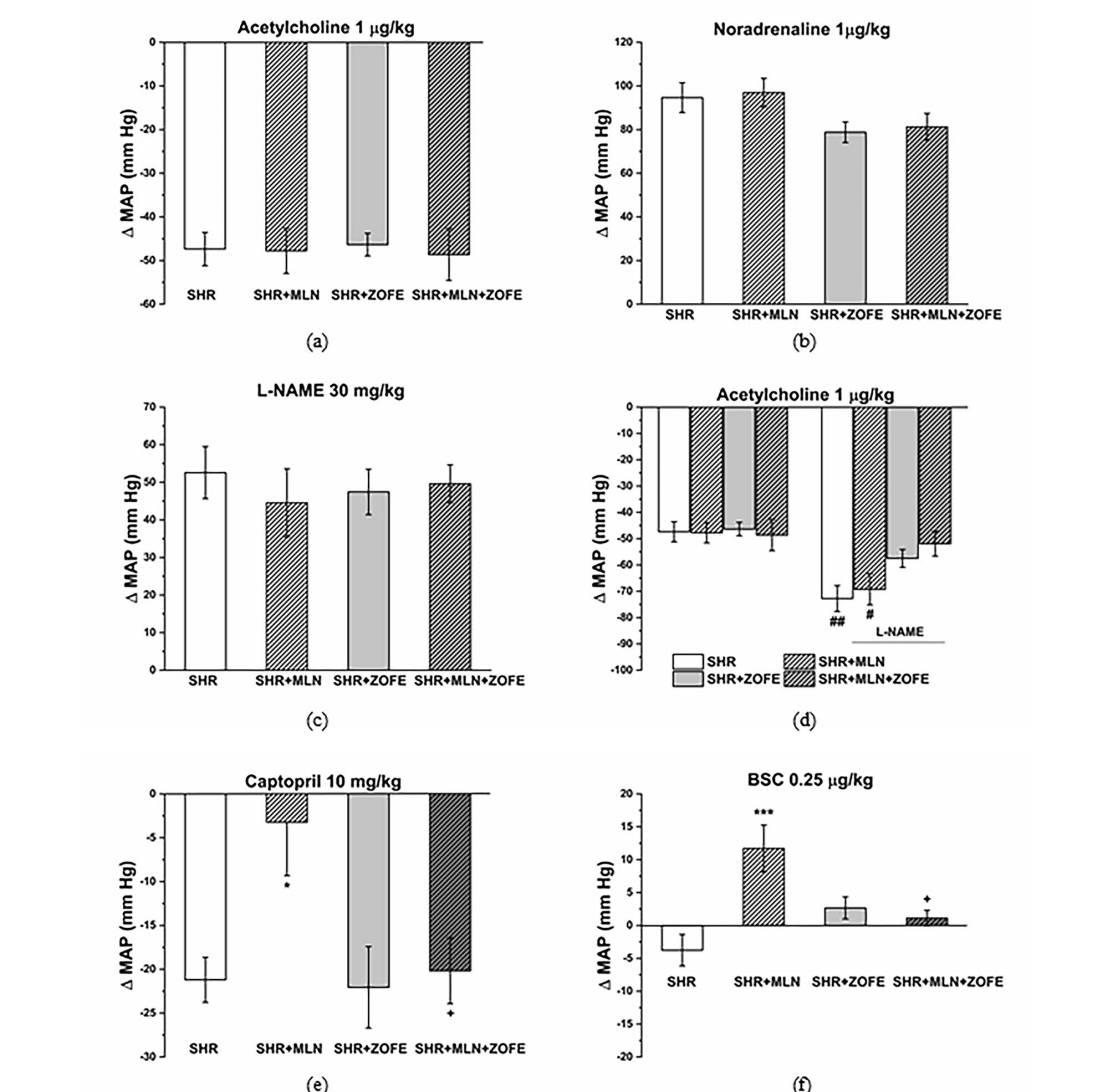
The effect of MLN and ZOFE treatment on the integrated pressure responses of the cardiovascular system. The changes in mean blood pressure (∆MAP) were induced by infusion of acetylcholine (1 µg/kg, **a**), noradrenaline (1 µg/kg, **b**), NOsynthase inhibitor (L-NAME, 30 mg/kg, **c**), ACE inhibitor captopril (10 mg/kg, **e**) and H_2_S scavenger bismuth(III) subsalicylate (BSC, 0.25 µg/kg, (**f**). The effect of acetylcholine (1 µg/kg) was also investigated before and after pretreatment with L-NAME (30 mg/kg, (**d**). SHR – spontaneously hypertensive rats (n = 8), SHR + MLN – spontaneously hypertensive rats treated by MLN-4760 (n = 8), SHR + ZOFE – spontaneously hypertensive rats treated by zofenopril (n = 8), SHR + MLN + ZOFE – spontaneously hypertensive rats treated by MLN-4760 and zofenopril (n = 8). The results are presented as the mean ± S.E.M., and differences between groups were analyzed by two-way ANOVA, *** p < 0.001 vs. SHR, ^+^ p < 0.05 vs. SHR + MLN, ^#^ p < 0.05, ^##^ p < 0.01 vs. before L-NAME

The basal values of MAP did not differ among the experimental groups at the beginning of the experiment (SHR: 96.6 ± 5.39 mmHg, SHR + MLN: 108.9 ± 4.01 mmHg, SHR + ZOFE: 101.8 ± 4.53 mmHg, SHR + MLN + ZOFE: 105.06 ± 6.85 mmHg). The bolus application of acetylcholine (1 µg/kg) evoked a decrease in the MAP, and this hypotensive response was comparable among the experimental groups (Fig. 3a). However, noradrenaline (1 µg/kg) induced a hypertensive response in rats, and neither the MLN nor the ZOFE and their combination influenced this response (Fig. 3b). Generally, i.v. application of N^G^-nitro-L-arginine-methyl ester (L-NAME, 30 mg/kg) increased the MAP in all groups, and the increase in MAP was comparable among the groups (Fig. 3c). L-NAME significantly increased the hypotensive response induced by acetylcholine in the SHR (p < 0.01) and SHR + MLN (p < 0.05) groups; however, in the SHR + ZOFE and SHR + MLN + ZOFE groups, the acetylcholine-induced responses before and after L-NAME application were comparable to the responses before the addition of L-NAME (Fig. 3d). Captopril (10 mg/kg) decreased the MAP in all groups; however, there was a significantly lower MAP decline in the MLN + SHR group (p < 0.05, Fig. 3e). However, whereas bismuth(III) subsalicylate (BSC, 0.25 µg/kg) induced a mild hypotensive response in the control SHR group, it induced a MAP increase in the SHR + MLN group (p < 0.001 vs. SHR). ZOFE treatment, however, reduced this elevation in MAP (p < 0.05 MLN ZOFE vs. MLN, Fig. 3f).

### Ex vivo studies

#### Relaxant responses of the thoracic aorta and the participation of NO, H_2_S and MasR pathways

**Fig. 4:**
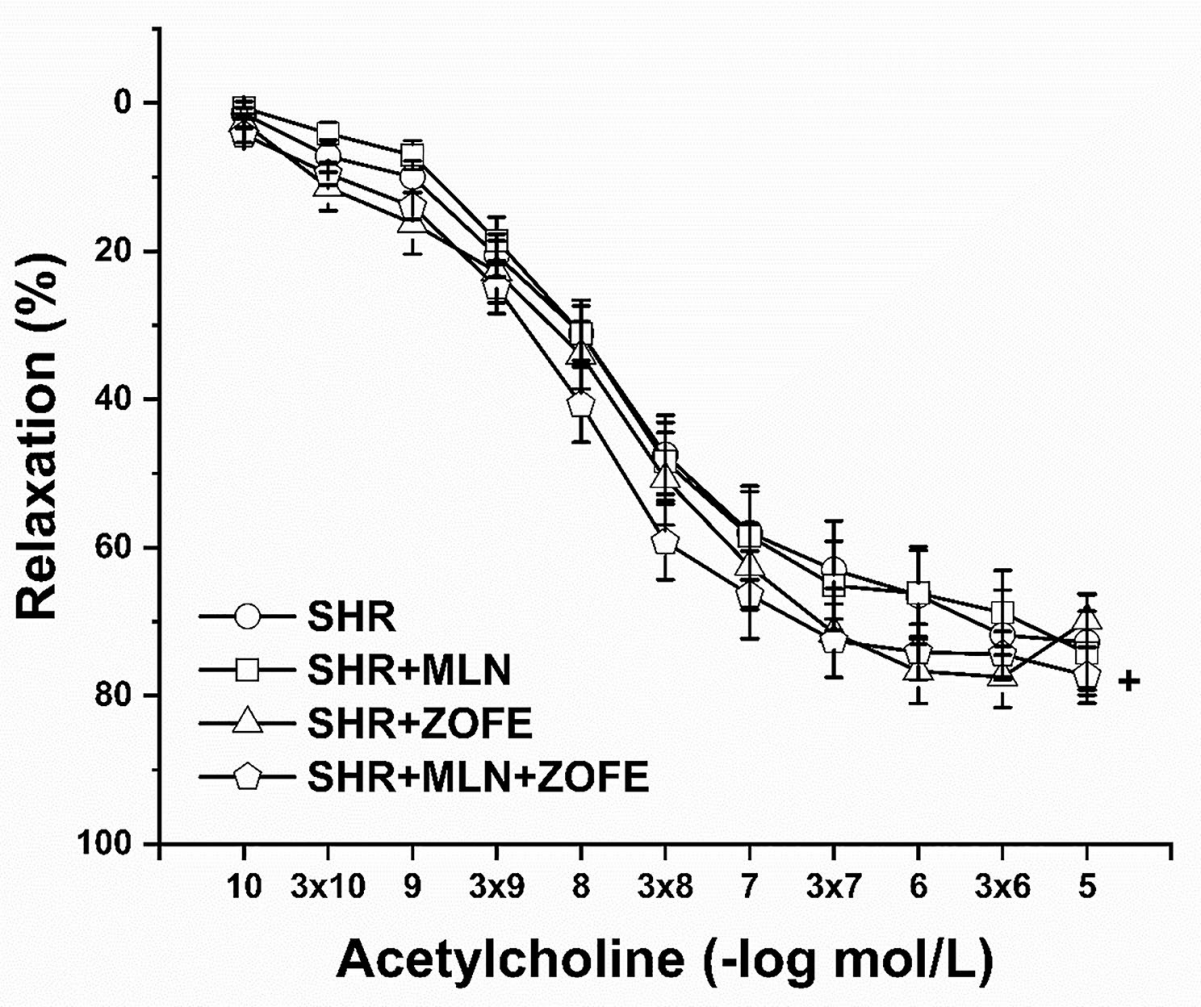
The effect of MLN and ZOFE treatment on the endothelium dependent vasorelaxation of the thoracic aorta isolated from spontaneously hypertensive rats (SHR, n = 10), spontaneously hypertensive rats treated by MLN-4760 (SHR + MLN, n = 10), spontaneously hypertensive rats treated by zofenopril (SHR + ZOFE, n = 10), spontaneously hypertensive rats simultaneously treated by MLN-4760 and zofenopril (SHR + MLN + ZOFE, n = 10). The results are presented as the mean ± S.E.M., and differences between groups were analyzed by three-way ANOVA, ^+^ p < 0.05 vs. SHR + MLN

Treatment with ZOFE had a significant effect on the endothelium-dependent vasorelaxation of TA (F_(1;627)_ = 17.47, p = 3.37 × 10^− 5^). ZOFE administration did not alter the relaxant responses compared to SHR, but in MLN-treated rats, it enhanced the relaxant capacity of TA (p < 0.05, SHR + MLN + ZOFE vs. SHR + MLN; Fig. 4).

**Fig. 5:**
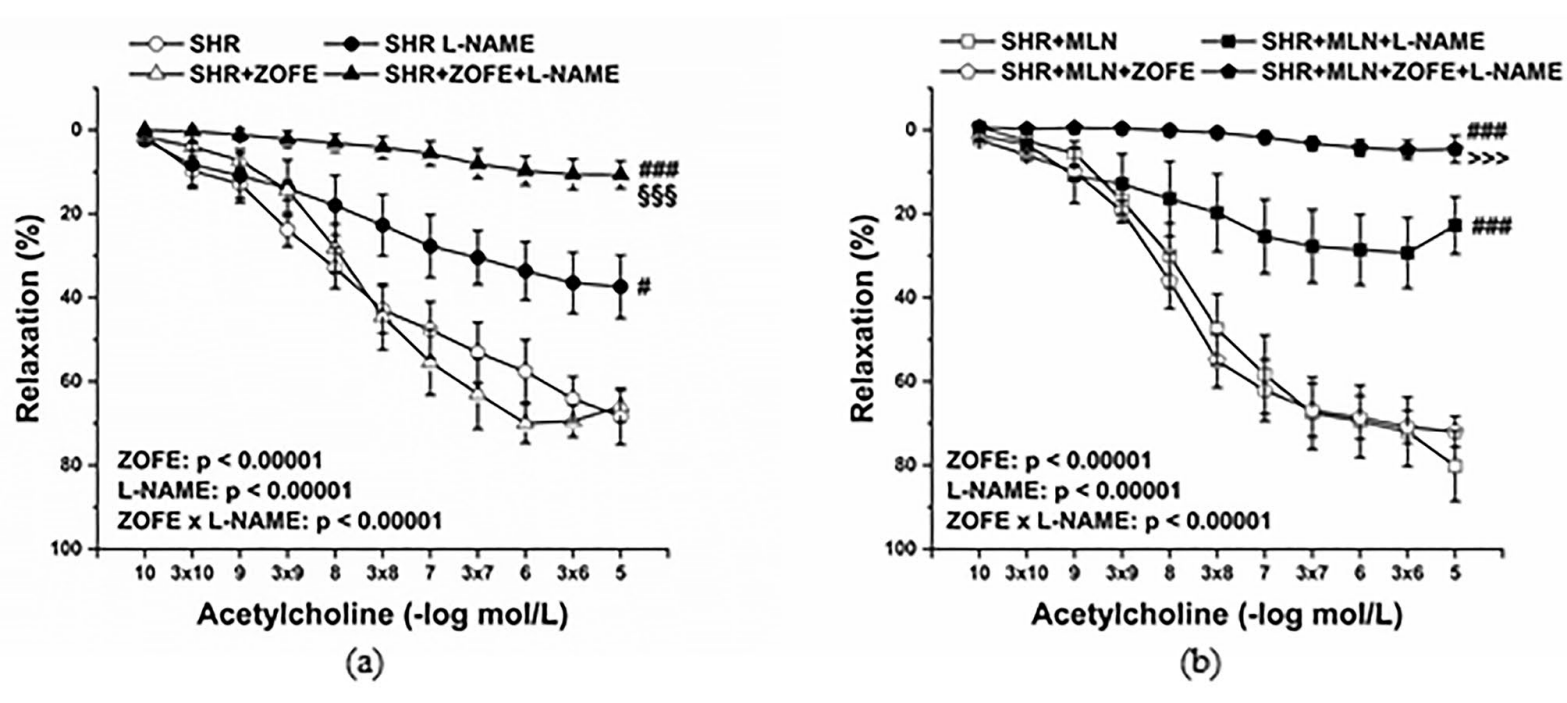
The effect of treatment with zofenopril on the participation of NO/NOS in endothelium dependent vasorelaxation in thoracic aortas isolated from rats without (**a**) or with (**b**) MLN-4760. SHR – untreated spontaneously hypertensive rats (n = 10); SHR + MLN – spontaneously hypertensive rats treated by MLN-4760 (n = 9); SHR + ZOFE – spontaneously hypertensive rats treated by zofenopril (n = 8); SHR + MLN + ZOFE – spontaneously hypertensive rats treated by MLN-4760 and zofenopril (n = 8). +L-NAME – aortic rings incubated with N^G^-nitro-L-arginine methylester. The results are presented as the mean ± S.E.M., and differences between groups were analyzed by three-way ANOVA, ^#^ p < 0.05, ^###^ p < 0.001 vs. the respective group without L-NAME, ^§§§^ p < 0.001 vs. SHR + L-NAME, ^>>>^ p < 0.001 vs. SHR + MLN + L-NAME

The participation of endogenous NO in vasorelaxant responses of TA was investigated via incubation of aortic rings with L-NAME (10^− 5^ mol/L). The effect of ZOFE treatment on NO participation in control SHRs is shown in Fig. 5a (effect ZOFE: F_(1;310)_ = 26.08, p = 6.24 × 10^− 7^, L-NAME: F_(1;310)_ = 232.22, p → 0, interaction of ZOFE x L-NAME: F_(1;310)_ = 32.53, p = 3.09 × 10^− 8^). Although acute incubation with L-NAME inhibited endothelium-dependent vasorelaxation in both the SHR and SHR + ZOFE groups, this inhibition was more pronounced in the SHR + ZOFE group than in the SHR group (p < 0.001). The extension of inhibition after L-NAME revealed a similar pattern in the SHR + MLN and SHR + MLN + ZOFE groups (Fig. 5b, effect ZOFE: F_(1;345)_ = 17.95, p = 3.02 × 10^− 5^, L-NAME: F_(1;345)_ = 321.88, p → 0, interaction of ZOFE x L-NAME: F_(1;345)_ = 29.68, p = 1.05 × 10^− 7^). In the presence of ZOFE, the inhibition was enhanced compared with the inhibition in the SHR + MLN group (p < 0.001).

**Fig. 6:**
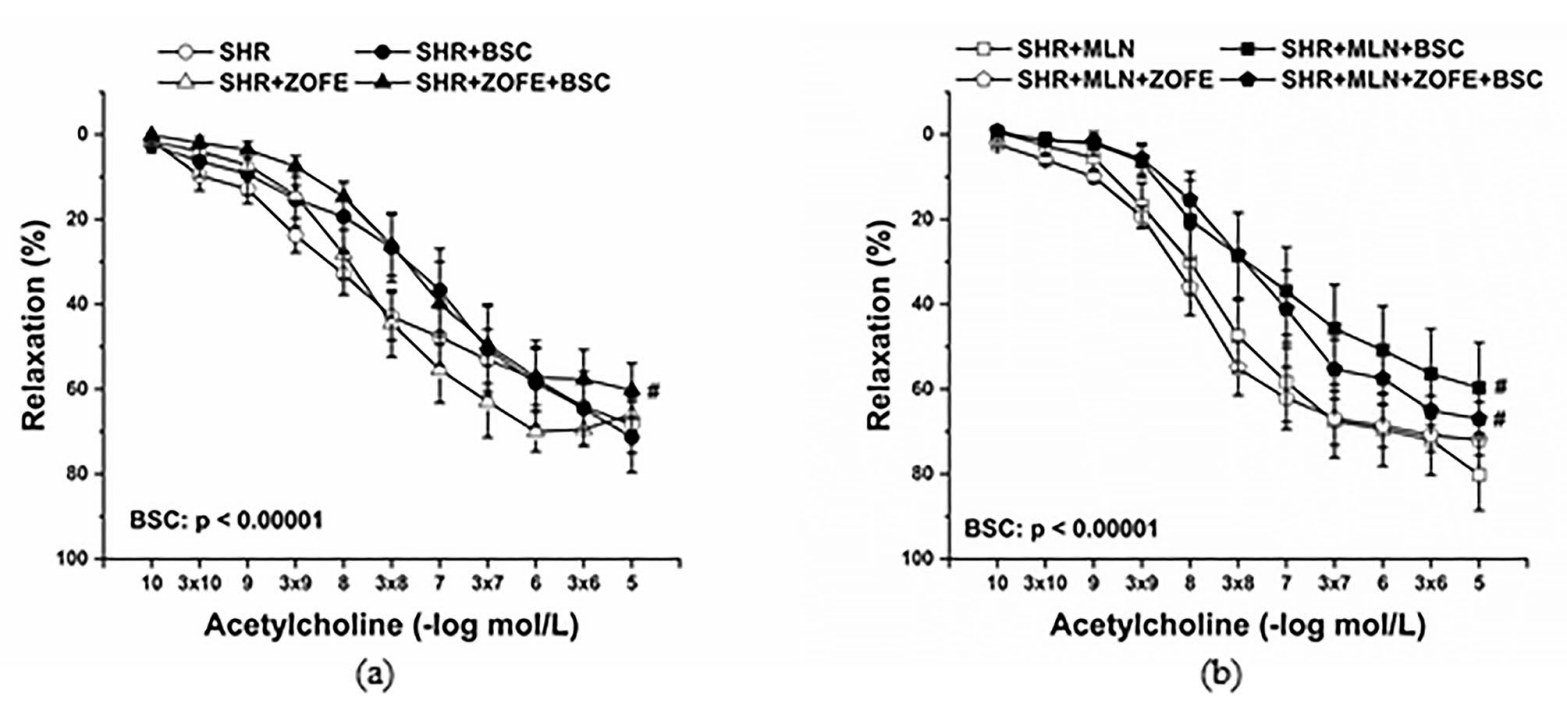
The effect of treatment with zofenopril on the participation of endogenous H_2_S in endothelium dependent vasorelaxation in thoracic aortas isolated from rats without (**a**) or with (**b**) MLN-4760. SHR – untreated spontaneously hypertensive rats (n = 10); SHR + MLN – spontaneously hypertensive rats treated by MLN-4760 (n = 9); SHR + ZOFE – spontaneously hypertensive rats treated by zofenopril (n = 8); SHR + MLN + ZOFE – spontaneously hypertensive rats treated by MLN-4760 and zofenopril (n = 8). +BSC– aortic rings incubated with bismuth(III) subsalicylate. The results are presented as the mean ± S.E.M., and differences between groups were analyzed by three-way ANOVA, ^#^ p < 0.05 vs. the respective group without BSC

The participation of endogenous H_2_S in the vasorelaxant responses of TA was investigated via incubation of aortic rings with BSC (10^− 6^ mol/L). Generally, incubation with BSC reduced endothelium-dependent relaxation in all experimental groups, except the SHR group (Fig. 6a, b; effect of BSC: F_(1;295)_ = 15.78, p = 9.29 × 10^− 5^, and F_(1;349)_ = 40.35), and ZOFE treatment had no effect on these vasoactive responses.

**Fig. 7:**
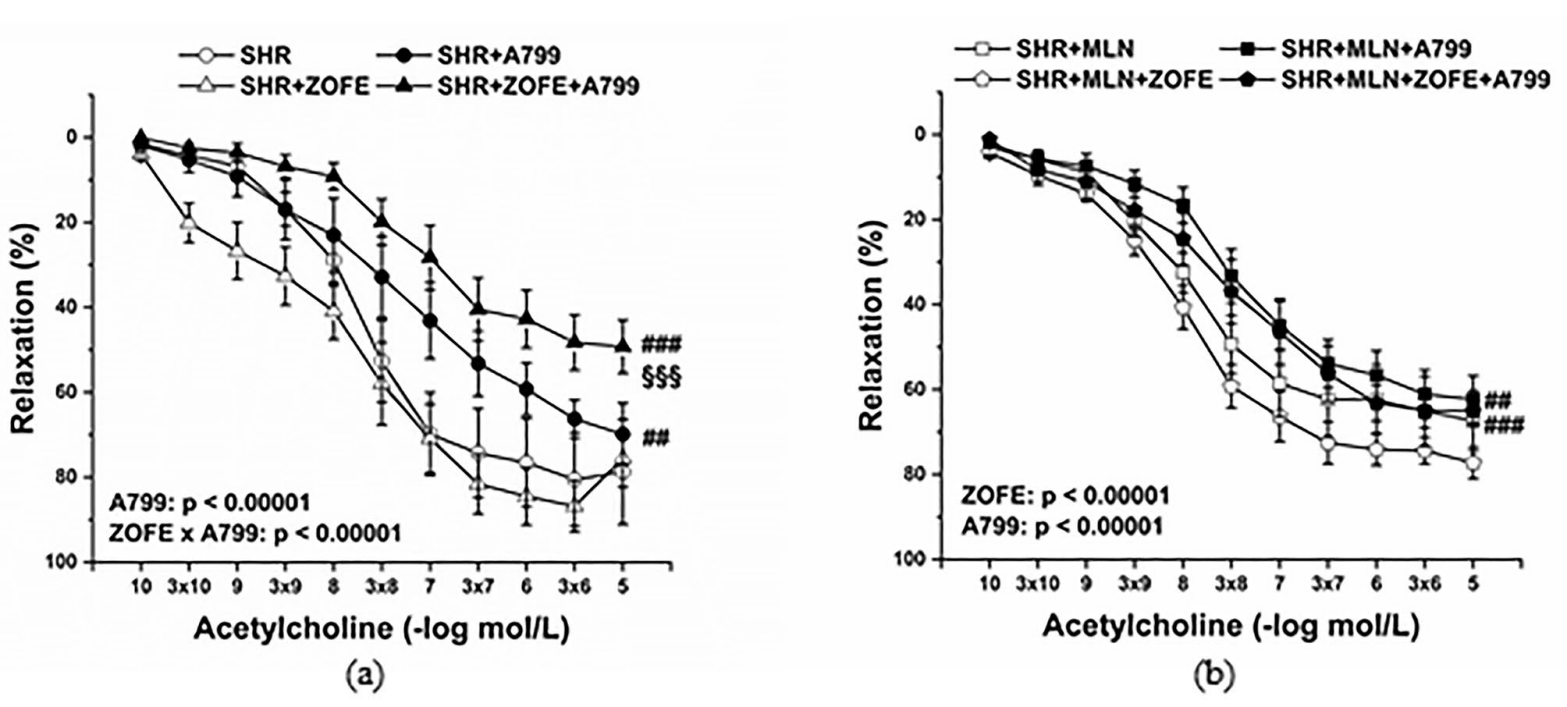
The effect of treatment with zofenopril on the participation of Mas receptors in endothelium dependent vasorelaxation in thoracic aortas isolated from rats without (**a**) or with (**b**) MLN-4760. SHR – untreated spontaneously hypertensive rats (n = 10); SHR + MLN – spontaneously hypertensive rats treated by MLN-4760 (n = 9); SHR + ZOFE – spontaneously hypertensive rats treated by zofenopril (n = 8); SHR + MLN + ZOFE – spontaneously hypertensive rats treated by MLN-4760 and zofenopril (n = 8). +A799 – aortic rings incubated with A-799 trifluoroacetate salt. The results are presented as the mean ± S.E.M., and differences between groups were analyzed by three-way ANOVA, ^##^ p < 0.01, ^###^ p < 0.001 vs. the respective group without A799p, ^§§§^ p < 0.001 vs. SHR + A799

To test the vasoactive manifestation of the Mas receptor-related pathway in the vasorelaxant responses of TA, the aortic rings were incubated with A-799 trifluoroacetate salt (A799; 10^− 5^ mol/L). Acute A799 incubation significantly reduced the relaxant response of TA in the SHR and SHR + ZOFE groups (Fig. 7a, p < 0.01 and p < 0.001, respectively, effect A799: F_(1;295)_ = 103.55, p →0 and interaction of ZOFE x A799: F_(1;195)_ = 26.82, 4.56 × 10^− 7^), and this inhibition was significantly enhanced in the SHR + ZOFE group compared with the SHR group (p < 0.001). However, A799 incubation blocked the vasorelaxation of TA in the SHR + MLN (p < 0.01) and SHR + MLN + ZOFE (p < 0.001) groups as well; this inhibition was comparable in these two groups (Fig. 7b).

#### Expression of selected proteins in left ventricle

**Fig. 8:**
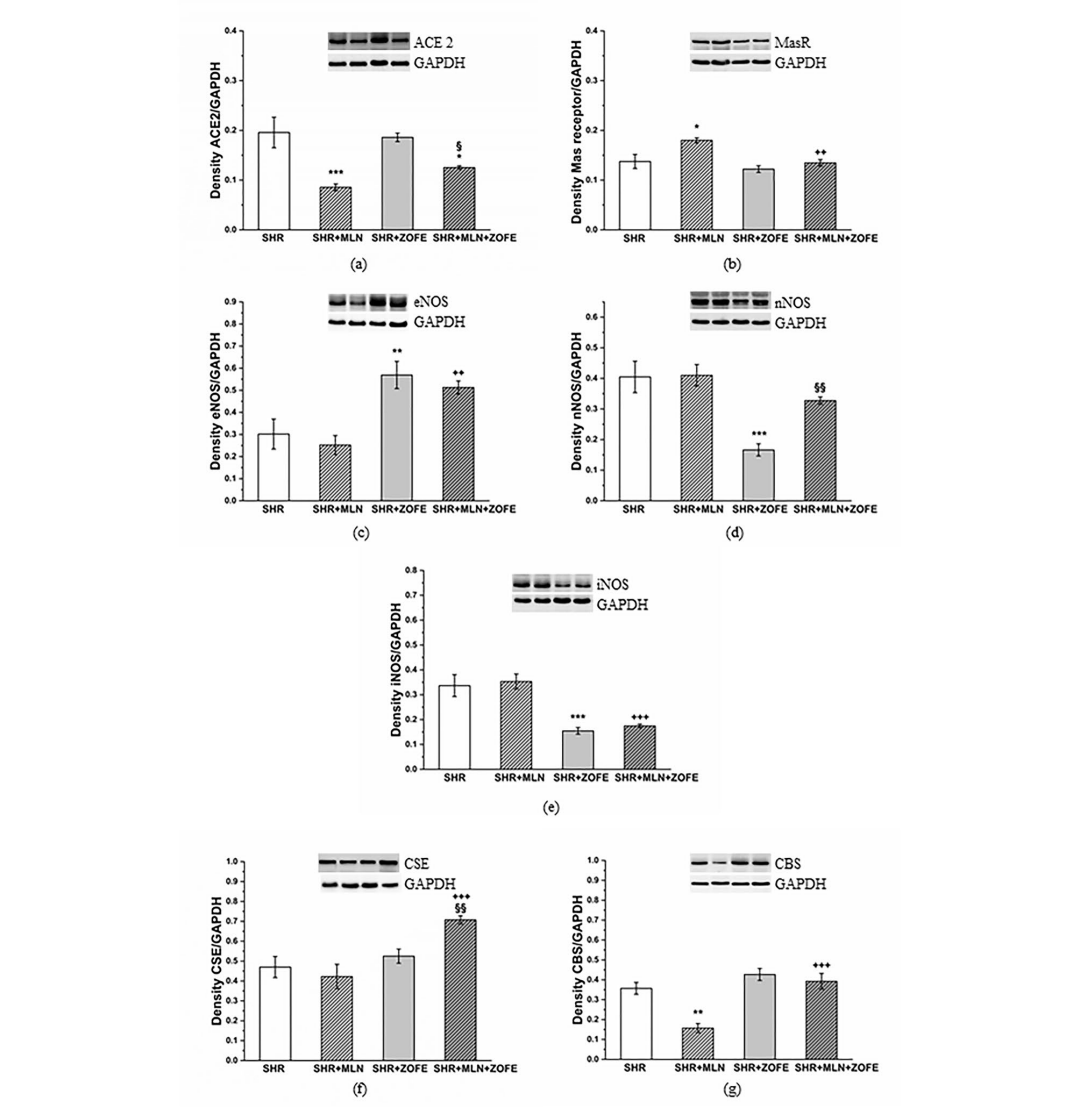
The protein expression of ACE2 (**a**), Mas receptor (**b**) eNOS (**c**) nNOS (**d**), iNOS (**e**), CSE (**f**) and CBS (**g**) in left ventricle isolated form spontaneously hypertensive rats (SHR, n = 8), spontaneously hypertensive rats treated by MLN-4760 (SHR + MLN, n = 8), spontaneously hypertensive rats treated by zofenopril (SHR + ZOFE, n = 8), spontaneously hypertensive rats treated by MLN-4760 and zofenopril (SHR + MLN + ZOFE, n = 8). The results are presented as the mean ± S.E.M., and differences between groups were analyzed by two-way ANOVA, * p < 0.05, ** p < 0.01, *** p < 0.001 vs. SHR, ^++^ p < 0.01, ^+++^ p < 0.001 vs. SHR + MLN, ^§^ p < 0.05, ^§§^ p < 0.01 vs. SHR + ZOFE

MLN treatment, regardless of ZOFE presence, reduced the protein expression of ACE2 (p < 0.001 vs. SHR, p < 0.05 vs. SHR + ZOFE, Fig. 8a) in the LV; however, MLN treatment induced an increase in the protein expression of the Mas receptor only in the SHR + MLN group (p < 0.05 vs. SHR, Fig. 8b). ZOFE administration and its simultaneous application with MLN increased the protein expression of endothelial NO synthase (eNOS) (p < 0.01) and reduced the expression of inducible NO synthase (iNOS) (p < 0.001, Fig. 8c, e). The expression of nNOS was reduced in the SHR + ZOFE group (p < 0.01); however, the simultaneous administration of MLN with ZOFE increased neuronal NO synthase (nNOS) expression (p < 0.01 vs. SHR + ZOFE, Fig. 8d). The expression of CSE was increased only in the SHR + MLN + ZOFE group (p < 0.001 vs. SHR + MLN, p < 0.01 vs. SHR + ZOFE), and CBS expression was reduced in the SHR + MLN group (p < 0.01 vs. SHR); however, this reduction was absent when MLN was applied in combination with ZOFE (p < 0.001 vs. MLN, Fig. 8f, g).

#### Expression of selected genes in aorta

**Fig. 9:**
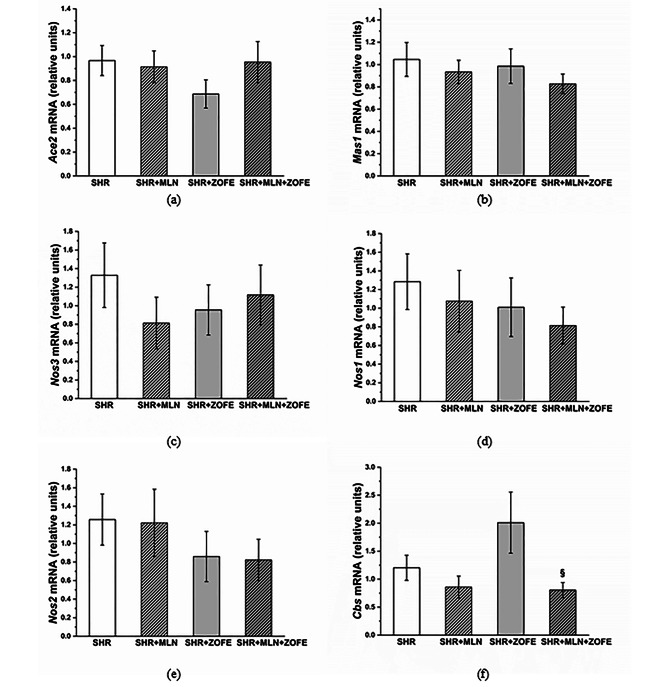
The mRNA levels of the *Ace2* (**a**), *Mas1* (**b**), *Nos3* (**c**), *Nos1* (**d**), *Nos2* (**e**) and *Cbs* (**f**) genes in aortic tissue isolated from spontaneously hypertensive rats (SHR, n = 8), spontaneously hypertensive rats treated by MLN-4760 (SHR + MLN, n = 8), spontaneously hypertensive rats treated by zofenopril (SHR + ZOFE, n = 8), spontaneously hypertensive rats treated by MLN-4760 and zofenopril (SHR + MLN + ZOFE, n = 8). The results are presented as the mean ± S.E.M., and differences between groups were analyzed by two-way ANOVA, ^§^ p < 0.05 vs. SHR + ZOFE

In the aorta, neither the ZOFE nor the MLN or their combination induced a significant effect on the mRNA levels of the *Ace2*, *Mas1*, *Nos3, Nos1*, and *Nos2* genes (Fig. 9a-e). We observed a decrease in *Cbs* gene expression in the SHR + MLN + ZOFE group compared to the SHR + ZOFE group (p < 0.05, Fig. 9f).

#### Expression of selected proteins in aorta

**Fig. 10:**
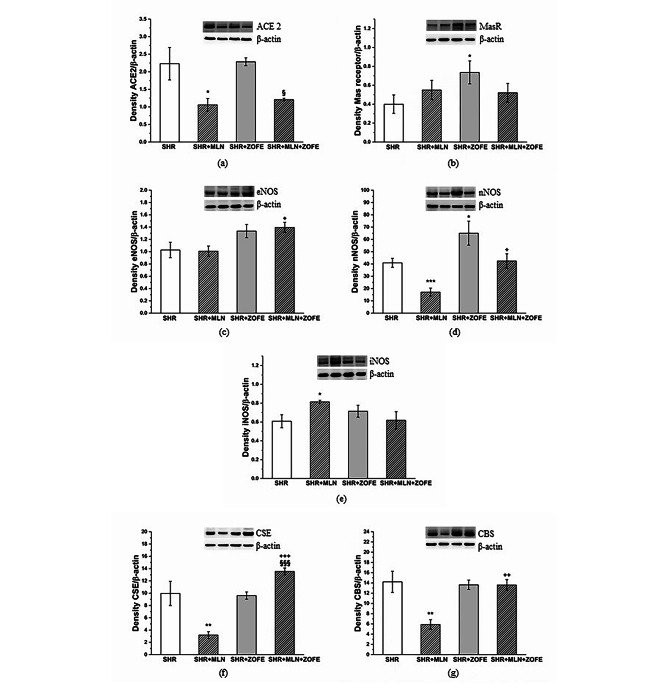
The protein expression of ACE2 (**a**), Mas receptor (**b**) eNOS (**c**) nNOS (**d**), iNOS (**e**), CSE (**f**) and CBS (**g**) in aortic tissue isolated form spontaneously hypertensive rats (SHR, n = 8), spontaneously hypertensive rats treated by MLN-4760 (SHR + MLN, n = 8), spontaneously hypertensive rats treated by zofenopril (SHR + ZOFE, n = 8), spontaneously hypertensive rats treated by MLN-4760 and zofenopril (SHR + MLN + ZOFE, n = 8). The results are presented as the mean ± S.E.M., and differences between groups were analyzed by two-way ANOVA, * p < 0.05, ** p < 0.01, *** p < 0.001 vs. SHR, ^++^ p < 0.01, ^+++^ p < 0.001 vs. SHR + MLN, ^§^ p < 0.05, ^§§§^ p < 0.001 vs. SHR + ZOFE

**Fig. 11:**
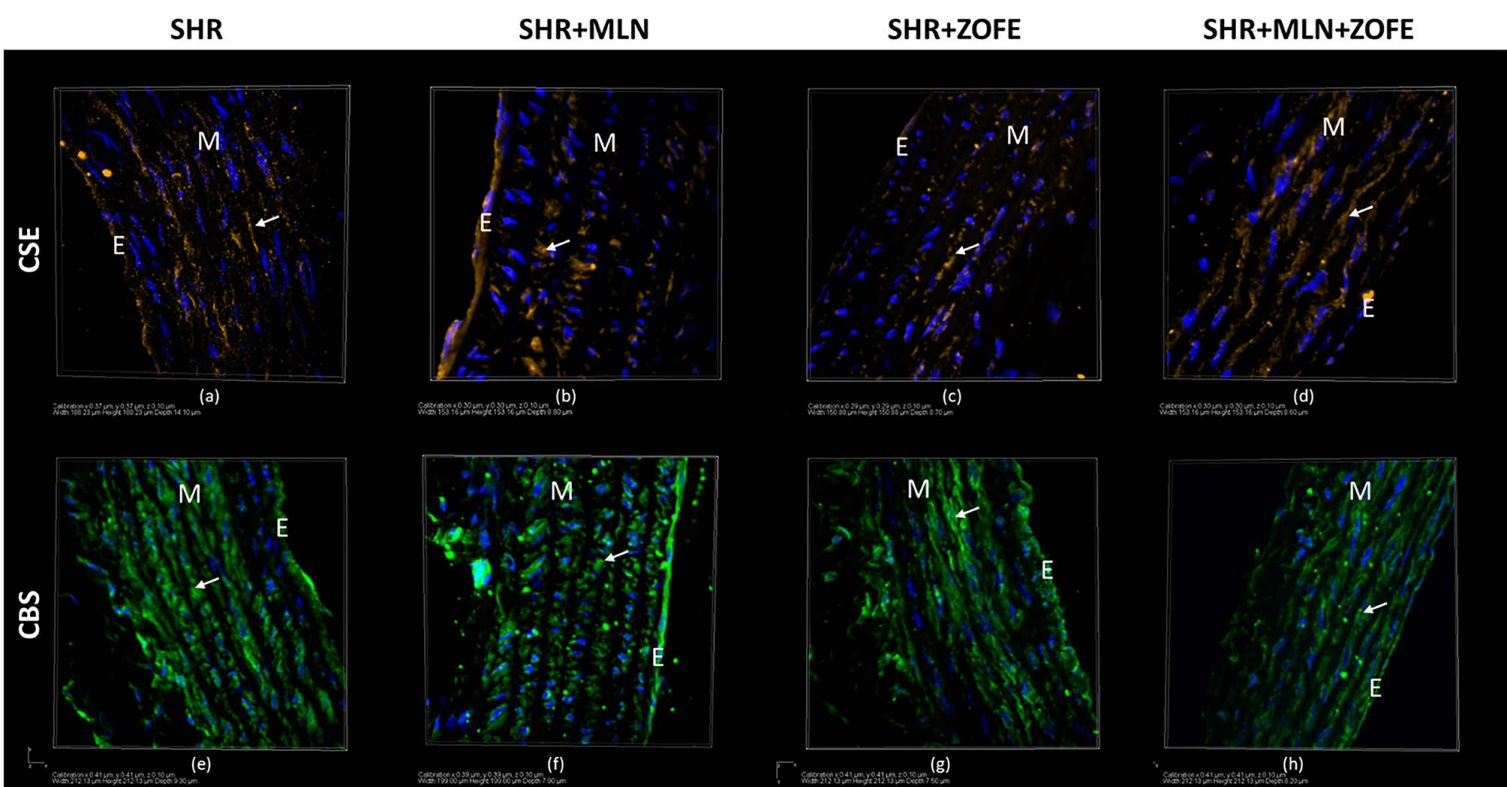
The distribution of CSE (orange color) and CBS (green color) enzymes shown using fluorescent stained of 10 μm thick cryosections of thoracic aorta tissue isolated from spontaneously hypertensive rats (SHR), spontaneously hypertensive rats treated by MLN-4760 (SHR + MLN), spontaneously hypertensive rats treated by zofenopril (SHR + ZOFE), spontaneously hypertensive rats treated by MLN-4760 and zofenopril (SHR + MLN + ZOFE). E – endothelium, M – tunica media. Blue color shows cell nuclei (stained by 4´,6´- diamidino-2-phenylindole, DAPI)

MLN treatment reduced the expression of ACE2 in the aorta even in the presence of ZOFE (p < 0.05); at the same time, the protein expression of Mas receptors was increased in the SHR + ZOFE group (p < 0.05, Fig. 10a, b). In the SHR + MLN + ZOFE group, there was an increase in eNOS expression compared to the eNOS expression in the SHR + MLN group (p < 0.05, Fig. 10c). While MLN administration decreased the expression of nNOS (p < 0.001), treatment with ZOFE evoked an increase compared to the SHR (p < 0.05) and SHR + MLN (p < 0.05) groups (Fig. 10d). The expression of iNOS was increased only in the MLN group (p < 0.01, Fig. 10e). The expression of both CSE and CBS was reduced after MLN administration (p < 0.01), and ZOFE alone had no impact on their expression; however, simultaneous application of MLN and ZOFE increased CSE expression compared with the CSE expression in the SHR + MLN (p < 0.001) and SHR + ZOFE (p < 0.001) groups and CBS expression compared with the CBS expression in the SHR + MLN group (p < 0.01, Fig. 10f, g). Figure 11 is an illustrative topography of H_2_S-producing enzymes in the thoracic aorta. Fluorescent staining confirmed the distribution of CSE and CBS mainly in the tunica media region in all investigated groups (Fig. 11).

#### Total NOS activity and superoxide production in the left ventricle and aorta

**Fig. 12:**
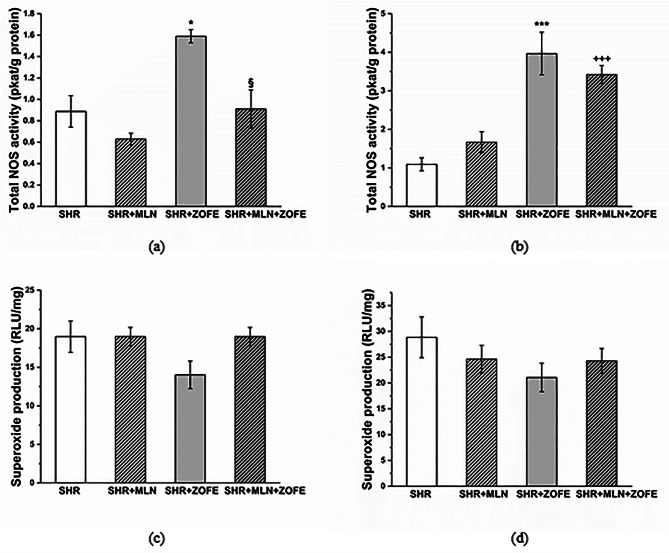
The total NOS activity and superoxide production in left ventricle (**a**,**c**) and aortic tissue (**b**,**d**). SHR – spontaneously hypertensive rats (n = 8), SHR + MLN – spontaneously hypertensive rats treated by MLN-4760 (n = 8), SHR + ZOFE - spontaneously hypertensive rats treated by zofenopril (n = 8), SHR + MLN + ZOFE - spontaneously hypertensive rats simultaneously treated by MLN-4760 and zofenopril (n = 8). The results are presented as the mean ± S.E.M., and differences between groups were analyzed by two-way ANOVA, * p < 0.05, *** p < 0.001 vs. SHR, ^+++^ p < 0.001 vs. SHR + MLN, ^§^ p < 0.05 vs. SHR + ZOFE

Treatment with ZOFE significantly increased the total NOS activity in the LV (p < 0.05) and aorta (p < 0.001). In the LV, the simultaneous administration of MLN and ZOFE reduced NOS activity compared with the NOS activity in the SHR + ZOFE group (p < 0.05). In aortic tissue, NOS activity remained increased in the SHR + MLN + ZOFE group as well (p < 0.001 vs. MLN, Fig. 12a, b). Neither ZOFE treatment alone nor its combination with MLN affected superoxide production in the LV and aorta (Fig. 12c, b).

## Discussion

In this study, we evaluated the influence of H_2_S-releasing ACEI ZOFE on cardiovascular functions in a rat model of human essential hypertension with altered ACE2 function. Previously, we showed a significant effect of treatment with the ACE2 inhibitor MLN on the increase in body weight, which was strengthened by significant negative correlations between body weight and visceral fat mass and between alternative RAS and Ang 1–7 plasma concentrations [6]. These results corresponded with previous findings of Bruce et al. [30], who found that activation of ACE2 resulted in reduced adiposity and body weight reduction. In this study, we found that ZOFE prevented the MLN-induced increase in BW and visceral fat-to-body weight ratio, suggesting the antiobesogenic action of ZOFE, in agreement with studies that revealed that ACE inhibition using ACEIs (e.g., captopril, perindopril) can lead to a reduction in BW as well as fat mass in different pathological conditions [31, 32]. However, the beneficial effect of ZOFE treatment on body adiposity did not go hand in hand with the effect on BP, inconsistent with the findings of Bucci et al. [15], who noted that administration of ZOFE at a dose of 10 mg/kg/day significantly lowered SBP in SHRs; however, after longer duration of ZOFE treatment. The insufficient effect of ZOFE on BP could be related to the presented plasma level of ANG II (Ang 1–8). Although we showed that MLN treatment alone had no effect on any of the angiotensin levels, ZOFE administration, alone or in combination with MLN, increased the levels of all analyzed angiotensins, including the level of ANG II. This surprisingly elevated level of ANG II might have several explanations. ZOFE administered at a dose of 10 mg/kg/day was previously shown to have decreased ACE activity in plasma by approximately 80% [33], in accordance with our results. The remaining activity of ACE could still be sufficient to convert increased levels of ANG I (Ang 1–10) to ANG II to even reach increased levels of ANG II. The above is strengthened by ZOFE increasing ANG I concentration approximately 4–5 times due to elevation of renin activity. The increase in Ang 1–7 seems to be insufficient to counterbalance ANG II since its absolute concentration is 10 times lower than the absolute concentration of ANG II. Another explanation could be associated with the chymase-mediated effect. Chymase, which is a chymostatin-sensitive ANG II-generating enzyme, produces ANG II independently of ACE, and Roszkowska-Chojecka et al. [34] confirmed that chymase contributed to ANG II-dependent hypertension in adult SHRs, thus suggesting that combined blockade of ACE and chymase-like enzymes could lower blood pressure more effectively than ACE inhibition alone. Additionally, SHR is a normal-to-low renin and normal-to-low angiotensin/aldosterone model of hypertension [23], whereas the sympathetic nervous system seems to be a dominant player in the development of hemodynamic, structural, and functional alterations in SHR [35]. Furthermore, the dominant knowledge on the effect of RAS-blockers is related to the circulating RAS system. However, the tissue-local RAS components do not always have to copy the level of plasmatic RAS and may participate in changes not corresponding to the circulating RAS. Finally, aldosterone may be an important player in cardiovascular alterations in animal models of hypertension [36].

As a next step, we focused on the evaluation of integrated blood pressure responses to vasoactive agents. We confirmed that the hypotensive response to acetylcholine and the participation of NO in blood pressure control remained unchanged after treatment with MLN. Acute NOS inhibition in both the SHR and SHR + MLN groups led to a more pronounced decline in the acetylcholine-induced hypotensive response; however, this effect disappeared after ZOFE administration regardless of ACE2 inhibition. The effect of ZOFE probably resulted from the properties of ACE inhibitors, which by decreasing bradykinin degradation can promote the release of NO. Comini et al. [37] declared that several ACE inhibitors (enalapril, perindopril, quinapril, ramipril, and trandolapril) increased eNOS protein expression and activity in the aorta and in cardiac myocytes as well as levels of circulating nitrite/nitrate, the end-metabolites of NO. Similarly, in our study, we also found a significantly increased activity of NOS in the aorta and in the LV after treatment with ZOFE. Another reason for the increased NO level after ZOFE treatment resides potentially in its increased bioavailability via reduced toxic free radical levels. If we assume that the acute inhibition of NO in the SHR and SHR + MLN groups might be partially or fully substituted by an increase in hyperpolarizing response to acetylcholine [38, 39], such a compensatory response was eliminated after repletion of NO due to ZOFE administration. Our suggestion about the role of bradykinin-mediated effects of ZOFE could also be proven by the results with captopril. The hypotensive response induced by the ACE inhibitor captopril was smaller in MLN-treated rats, suggesting the contribution of ACE2/Ang 1–7/alamadin-mediated pathways to the antihypertensive effect of captopril. Given that Ang 1–7, acting through Mas receptors, activates the release of tissue kallikrein-kinins and provides cardiovascular protection through bradykinin receptor-mediated effects [16], we hypothesize that MLN- treatment led to a disruption of this protective effect and reduced the hypotensive effect of captopril, which, however, was recovered after ZOFE treatment. The interplay between RAS and H_2_S signaling could also be considered in blood pressure control. In our study, we demonstrated that after acute addition of H_2_S scavenger (BSC), the mild hypotensive response observed in control rats changed to a significant blood pressure increase in rats treated with MLN, suggesting considerable changes in H_2_S levels resulting in switching of H_2_S action from vasoconstrictor to vasorelaxant. The literature shows that low concentrations of H_2_S induce vasoconstriction and higher concentrations lead to vasorelaxation [40], which corresponds with our finding of increased concentrations of H_2_S in plasma and heart after treatment with MLN. However, when ZOFE was coadministered with MLN, the plasma H_2_S level as well as its level in cardiac tissue remained elevated, but the vasodilatory effect of H_2_S was reduced (the addition of BSC reduced the vasopressor response). The reason for this discrepancy is unknown, but since blood pressure responses reflect the interplay between cardiac output and total peripheral vascular resistance, one can speculate that the concentration of H_2_S and vasodilator effect in the resistant part of arterial tree could have been reduced.

Regarding the effects of ZOFE treatment on the heart, we confirmed its significant impact on systolic and diastolic cardiac function. In SHR, pathological myocardial fibrosis associated with hypertension develops together with left ventricular (LV) systolic and diastolic dysfunction [41]. Our results showed that MLN treatment did not affect the LV function of SHRs, which was already significantly afflicted in SHRs compared to controls in our previous experiment [23]. However, treatment with ZOFE, alone or in combination with an ACE2 inhibitor, led to a beneficial increase in LV fractional shortening and ejection fraction as well as to improvement in diastolic function. ZOFE, due to its high lipophilicity, could potentially easily penetrate cardiac tissue and exert its antiremodeling effect regarding attenuation of LV hypertrophy, fibrosis, and LV functional improvement in SHR [27, 42, 43]. ZOFE was also reported to stimulate active Ca^2+^ uptake via the sarcoplasmic reticulum cycle in cardiomyocytes, which could account for the improvement in myocardial contractility after ischemia-reperfusion damage [44] and improvement of diastolic relaxation in our experiment. Nevertheless, Donnarumma et al. [45] confirmed that ZOFE-mediated cardioprotection in murine and swine models of myocardial ischemia/reperfusion injury was associated with an increase in H_2_S levels, and Bucci et al. [15] confirmed that the release of H_2_S from zofenopril, an active metabolite of S-zofenopril, represented an additional beneficial mechanism unrelated to ACE inhibition. Generally, H_2_S was shown to be a potent cardioprotective signaling molecule that significantly attenuated the pathological consequences of myocardial ischemia/reperfusion injury and heart failure and ameliorated LV remodeling and cardiac fibrosis in SHR [46]. Moreover, it seems that ZOFE can not only release H_2_S but also influence its endogenous production. In our experiment, we found that treatment with ZOFE in MLN-treated rats led to increased expression of H_2_S-producing enzymes in the LV, corresponding with findings that H_2_S donors can stimulate the expression of H_2_S-producing enzymes in the myocardium during various pathological conditions. Li et al. [47] demonstrated that treatment with H_2_S donors increased CBS levels and protected cardiomyocytes from hypoxic injury. Pan et al. [48] indicated that NaHS treatment increased CSE protein expression and prevented cardiac remodeling in postmyocardial infarction rats. We found an increased level of H_2_S in heart tissue after MLN treatment; however, the increased level was not associated with the improvement in cardiac function. We suppose that the beneficial effects of enhanced sulfide signaling could be masked by deleterious effects of ACE2 inhibition on the MasR/NO signaling cascade. Tan et al. [49] confirmed that cardiac gene and protein expression of ACE2 and Mas receptors is significantly decreased in SHR compared to normotensive rats. In this study, we showed that administration of an ACE2 inhibitor led to a further decrease in ACE2 protein expression in the LV, which, in turn, was compensated by an increase in the expression of Mas receptors; however, it was apparently not sufficient to increase NO levels and NO-associated signaling, which represent another beneficial mechanism improving cardiac function. In contrast, after treatment with ZOFE, similar to our study, Donnarumma et al. [45] reported increased levels of cardiac NO due to increased phosphorylation of eNOS at the Ser1177 site, which promotes eNOS activation. Moreover, nNOS also plays an important role in the autocrine control of cardiac contractility by regulating Ca^2+^ fluxes. Sears et al. [50] suggested that nNOS-derived NO regulated myocardial contraction and Ca^2+^ handling in cardiomyocytes of the LV, thereby protecting against Ca^2+^ overload in cardiac disease states. In the hypertrophic LV of SHR, Piech et al. [51] found preserved NOS activity, which was mediated by increased expression of nNOS protein as a compensatory response to the decrease in eNOS. These data agree with our findings after administration of ZOFE. In both groups treated with ZOFE, we demonstrated increased NOS activity in the LV, which corresponded to an increase in eNOS expression. However, nNOS expression was reduced after ZOFE administration to control SHR, which can be considered a feedback mechanism having in mind the excess of nNOS. In the group where ZOFE was administered together with MLN, such a decline was not observed; moreover, the activity of NOS was reduced compared to the activity in the ZOFE group. Considering a complex interaction between NO and H_2_S [52], we suppose that the strengthened sulfide and nitroso pathways could underlie the beneficial effect of ZOFE on LV function; however, the administration of an ACE2 inhibitor could partly attenuate the impact of NO signaling.

The aorta plays an important role in the control of systemic vascular resistance and heart rate, in addition to its conduit function. Therefore, we determined the vasoactive properties of the thoracic aorta and simultaneously investigated the importance of individual signaling pathways involved in its relaxation function. The thoracic aorta, as a type of elastic artery, undergoes specific structural and functional remodeling during hypertension, and the hypertrophied arterial wall displays decreased contractile efficiency [53]. Moreover, the thoracic aorta of SHRs may exhibit partial endothelial dysfunction, but this dysfunction could be mediated by other factors (such as overproduction of cyclooxygenase-derived vasoconstrictors or superoxide) rather than by an inhibited NO signaling pathway [53]. In our previous study, we showed that the endothelium-dependent relaxation of the thoracic aorta remained unchanged, whereas NO signaling was stimulated (or at least fully functional) after treatment with an ACE2 inhibitor, probably due to activation of compensatory vasoactive mechanisms [6]. In this study, we revealed that ZOFE administration did not alter the relaxant responses compared to control SHR, but in MLN-treated rats, ZOFE administration enhanced the relaxant capacity. To examine whether the potentiation of relaxation was mediated by NO, we inhibited the endogenous NO production by L-NAME, and we confirmed that ZOFE increased the NO component of endothelium-dependent vasorelaxation equally, regardless of MLN treatment. This increase was associated with increased NOS activity and unchanged superoxide production in both groups. Based on protein analyses of NOS isoforms, in control SHR, nNOS was responsible mainly for the increased total NOS activity after ZOFE treatment, although there was also a trend toward increased expression of the others. However, MLN treatment can alter the balance among the individual NOS isoforms and antagonize the effect of ZOFE. We observed that protein expression of nNOS decreased after treatment with MLN in the aorta, in parallel with reduced ACE2 expression. This agrees with the finding of Zheng et al. [54], who confirmed that an increase in ACE2 expression caused an increase in nNOS expression in neuronal cell lines, which could also work vice versa. Conversely, the expression of iNOS was increased after MLN, similar to the findings of Cheng et al. [55], who suggested that upregulation of iNOS in the aortas of SHRs could be a compensatory mechanism and not only a response to cellular stress. Nevertheless, proinflammatory cytokines such as tumor necrosis factor alpha (TNF-α) and interleukin-1 (IL-1) are the main inducers of iNOS expression via the cascade of kinase activation and phosphorylation of specific transcription factors, including nuclear factor κB [56]. However, ZOFE has protective effects, including the capability of scavenging ROS, and it can also suppress the inflammatory effect by increasing the activity of the antioxidant enzymes SOD, CAT and GSH, thus decreasing the expression of iNOS [57]. After administration of ZOFE in MLN-treated rats, the expression of iNOS returned to the control level (aorta) or decreased (heart), and increased NOS activity was mediated mainly through increased expression of eNOS. Moreover, ZOFE altered the expression of individual NOS isoforms in the aorta and heart differently. All these effects of ZOFE could be associated with tissue-dependent regulation of NOS isoforms in SHR, as shown by Piech et al. [51]. Authors demonstrated that, compared to normotensive rats, eNOS abundance increases in the aorta of 18 weeks SHR, whereas its level decreases in the LV. However, the expression of nNOS increased in the LV of SHR, but it was unchanged in the aorta. Interestingly, there were no changes in the gene expression of *Nos1*, *Nos2*, *Nos3* or *Mas1* and *Ace2* in the aorta, suggesting that the majority of MLN and/or ZOFE effects occurred posttranscriptionally. Altogether, our results showed that ZOFE stimulated NO signaling in both the heart and aorta, although the individual NOS isoforms were altered differently. Such diversity highlights the need to investigate the regulation of individual NOS isoforms to gain further understanding of their functional impact on downstream NO signaling.

Another mechanism that could be involved in the potentiated relaxant capacity in the aortas of MLN + ZOFE-treated rats could be associated with accentuation of the H_2_S-mediated functional impact. Incubation with the H_2_S scavenger reduced endothelium-dependent relaxation in all experimental groups, except for the control SHR, and ZOFE treatment had no effect on these vasoactive responses, suggesting that H_2_S participated in maintaining endothelial function in MLN-treated rats, which could be considered a compensatory mechanism. This observation corresponds with other findings that declare that H_2_S signaling can be stimulated in different pathological conditions, e.g., in fructose-fed normotensive rats [58] as well as in SHRs, where the increased participation of H_2_S in vasorelaxation could counterbalance endogenous NO deficiency [53]. The decreased expression of H_2_S-producing enzymes in the MLN-treated rats could be explained by negative feedback regulation to maintain a constant H_2_S level. After treatment with ZOFE, the protein expression of H_2_S-producing enzymes returned to the control level, probably due to the restoration of the balance in relaxation factors (as the production of H_2_S did not increase further). Taken together, our findings showed that ZOFE itself can stimulate H_2_S signaling, as found by other authors [14, 15], but the potentiation of this pathway was not responsible for elevated relaxation during ACE2 inhibition. The further signaling pathway under our investigation was the pathway of Mas receptors. Silva et al. [59] demonstrated that an exercise-induced increase in Mas receptor expression in SHR aortas improved Ang 1-7-mediated vascular function, acting as a counter regulator of classic Ang II/AT1-mediated effects. Similarly, Zhang et al. [60] showed that stimulation of Mas receptors by Ang 1–7 not only improved endothelium-dependent vasorelaxation but also inhibited contractile responses in SHR. In this study, ZOFE increased vasorelaxant effects mediated by Mas receptor signaling; however, simultaneous treatment with MLN eliminated this potentiating effect, which corresponded with changes in the protein expression of Mas receptors and ACE2. We can conclude that although the increased participation of the Mas receptor-mediated pathway could be triggered by ZOFE, the concomitant inhibition of ACE2 might result in activation of other mechanisms initiating a stronger vasorelaxation response which could be associated, for example, with almandine, AcSDKP or Ang II-AT2 receptor-mediated protective pathways.

### Limitations

There are several potential limitations of this study. The use of an animal model and evaluation of selected parameters does not allow to replicate all aspects of the human disease. The choice of the experimental model, the selection of doses of both inhibitors, as well as the duration of their application could have an impact on the achieved results. SHR model was chosen based on the declared similarity with human essential hypertension. One should consider that other frequently used experimental models with different pathological mechanisms of hypertension development, such as L-NAME- or angiotensin II- induced hypertension, might produce different results. In addition, the potentially escalated metabolic disorder due to the longer-lasting action of ACE2 inhibitor could slow-down or even suppress the onset of compensatory vasoactive mechanisms associated with the activation of the sulfide signaling pathway [61]. Finally, the study did not evaluate the influence of inhibitors on selected signaling pathways in small and resistant arteries, which could significantly affect the vascular tone and blood pressure control. Evaluation of vasoactive responses and monitoring of signaling pathways in these arteries would be appropriate to add in the future to create a more complex view.

## Conclusions

In conclusion, our study revealed that treatment with zofenopril reduced MLN-induced adiposity and improved the cardiac function of SHRs independently of ACE2 inhibition. Moreover, the concomitant MLN and zofenopril treatment increased thoracic aorta vasorelaxation capacity, although zofenopril increased the participation of H_2_S and NO in the maintenance of endothelial function independently from ACE2 inhibition. These new findings confirmed that the beneficial effects of zofenopril were not affected by ACE2 inhibition and that using of ACEIs could be recommended therapy even during ACE2 pathway disruption. Moreover, the second important output is that ACE2 inhibition itself could trigger cardiovascular compensatory pathways associated with activation of cardiac Mas receptors, increased sulfide plasmatic and cardiac level, and stimulation of arterial nitroso pathway.

## Data Availability

The data used to support the findings of this study are available from corresponding author upon request.

## Abbreviations

A: The diastolic transmitral peak late filling velocities
ACE: Angiotensin-converting enzyme
ACE2: Angiotensin-converting enzyme 2
ACEI: Angiotensin-converting enzyme inhibitor
ACE-S: Soluble angiotensin converting enzyme
Ach: Acetylcholine
ALT-S: Total alternative renin angiotensin system activity
Am: Late diastolic wall movement waves
Ang: Angiotensin
ARBs: Angiotensin receptor I blockers
BSC: Bismuth(III) subsalicylate
BW: Body weight
CBS: Cystathionine-β-synthase
CSE: Cystathionine-γ-lyase
E: The diastolic transmitral peak early filling velocities
Em: Early diastolic wall movement waves
H_2_S: Hydrogen sulfide
HW: Heart weight
L-NAME: N^G^-nitro-L-arginine methyl ester
LV: Left ventricle
LVEF: Left ventricle ejection fraction
LVFS: Left ventricle fractional shortening
MAP: Mean arterial pressure
NA: Noradrenaline
NOS: NOsynthase
Nos1: Neuronal NOsynthae
Nos2: Inducible NOsynthase
Nos3: Endothelial NOsynthase
PRA: Plasma renin activity
RAS: Renin-angiotensin system
SARS-COV-2: Severe acute respiratory syndrome coronavirus 2
SBP: Systolic blood pressure
SHR: Spontaneously hypertensive rats
TA: Thoracic aorta
VF: Visceral fat
ZOFE: Zofenopril
